# Duration of Induced Hypothermia After Pediatric Cardiac Arrest: A Systematic Review and Network Meta-Analysis

**DOI:** 10.3390/jcm15218364

**Published:** 2026-10-27

**Authors:** Jinpyo Kim, Tae Nyoung Chung

**Affiliations:** Department of Emergency Medicine, CHA Bundang Medical Center, CHA University School of Medicine, Seongnam 13496, Republic of Korea; a256096@chamc.co.kr

**Keywords:** pediatric cardiac arrest, hypothermia, treatment duration, temperature management, network meta-analysis, survival, neurologic outcome

## Abstract

**Background/Objectives**: Pediatric guidelines retain induced hypothermia as an option, but its optimal duration is uncertain. We compared survival and favorable neurologic outcomes across duration-specific strategies. **Methods**: Five databases and ClinicalTrials.gov were searched through 20 August 2026. A random-effects network meta-analysis compared 24, 48, and 72 h hypothermia (HT) with heterogeneous Control strategies. Risk of bias was assessed using the Cochrane Risk of Bias 2 tool and the Risk Of Bias In Non-randomized Studies of Interventions tool; certainty was assessed using Confidence in Network Meta-Analysis (CINeMA). **Results**: Survival included five studies (three randomized, two observational; 785 participants), and neurologic outcomes included four (three randomized, one observational; 615 participants). HT-72 h was associated with higher survival than Control (risk ratio [RR], 2.01; 95% confidence interval [CI], 1.24–3.25); 72% of this estimate derived from one observational study measuring 1-month survival, versus 12 months in the randomized 48 h trials. The estimate versus HT-48 h (RR, 1.77; 95% CI, 1.05–2.97) was entirely indirect and depended on merging different Control strategies. The neurologic estimate versus Control (RR, 10.92; 95% CI, 1.32–90.40) depended on a single observational study at serious risk of bias and was extremely imprecise. Superiority over shorter durations was not robust. Certainty for HT-72 h versus Control was very low for both outcomes. **Conclusions:** Evidence remains compatible with equipoise and does not establish an optimal duration. **Funding:** No external funding. **Registration: Open Science Framework,** 10.17605/OSF.IO/9MNDB (retrospective).

## 1. Introduction

Pediatric cardiac arrest is uncommon but is associated with high mortality and substantial long-term neurologic morbidity among survivors [1,2,3]. Temperature management is therefore a central component of pediatric post-cardiac arrest care. Current pediatric resuscitation guidelines recommend active temperature management and include induction of hypothermia at 32–34 °C as a treatment option alongside active normothermia and fever prevention [1,2]. However, they do not establish the optimal duration of the hypothermic phase. The principal randomized evidence came from the Therapeutic Hypothermia After Pediatric Cardiac Arrest (THAPCA) trials in out-of-hospital cardiac arrest (OHCA; THAPCA-OH) and in-hospital cardiac arrest (IHCA; THAPCA-IH), which compared 48 h of hypothermia followed by active normothermia with active normothermia alone [4,5]. Although the conventional frequentist analyses did not demonstrate statistically significant superiority, a subsequent Bayesian reanalysis of THAPCA-OH estimated a 94% probability that the hypothermia strategy provided some benefit for both 1-year survival with favorable neurobehavioral outcome and 1-year survival [6]. These findings preserve clinical interest in induced hypothermia but do not determine whether 48 h, a shorter course, or a longer course is preferable.

Evidence directly comparing hypothermia durations remains sparse. The evolving window of secondary brain injury makes duration a plausible effect modifier [3]. A pilot randomized trial compared 24 and 72 h of hypothermia and found lower concentrations of selected brain-injury biomarkers after longer cooling but was not powered to establish differences in clinical outcomes [7]. Other duration-specific evidence consists largely of observational studies using approximately 24 or 72 h protocols in populations that differed by arrest setting, etiology, severity, resuscitation duration, comparator temperature strategy, and outcome timing [8,9,10,11]. Consequently, neither individual studies nor a simple comparison of hypothermia with normothermia can determine the optimal duration of the hypothermic phase. Longer cooling is not necessarily better: 120 versus 72 h did not improve neonatal intensive care survival in hypoxic–ischemic encephalopathy, and 48 versus 24 h did not significantly improve neurologic outcomes after adult cardiac arrest [12,13]. These populations are not directly interchangeable with pediatric cardiac arrest.

Previous pairwise meta-analyses generally combined hypothermic protocols of different durations into a single intervention category. Buick et al. found no clear evidence of benefit or harm [14], whereas Alsabri et al. reported favorable associations after pediatric OHCA but judged the evidence to be of low certainty [15]. A prior network meta-analysis (NMA) separated hypothermia by duration and ranked 72 h most favorably [16]; however, it pooled the potentially overlapping Lin 2013 [10] and 2018 [11] reports and classified the variable-duration Fink 2010 [9] cohort as fixed 24 h evidence. A newer pragmatic registry study has since expanded the evidence base [17]. The ongoing Pediatric Influence of Cooling Duration on Efficacy in Cardiac Arrest Patients (P-ICECAP) adaptive randomized trial directly addresses pediatric cooling duration but has not yet reported outcomes [18]. An updated analysis was therefore needed to distinguish fixed intended-duration evidence from approximate or pragmatic exposure and to test whether duration-specific findings were robust to study classification and overlapping report selection.

We conducted an updated systematic review and NMA to compare 24, 48, and 72 h hypothermia strategies after pediatric cardiac arrest with respect to survival and long-term favorable neurologic outcome. Primary analyses used fixed intended-duration evidence, while sensitivity analyses evaluated approximate-duration evidence and an alternative potentially overlapping report. We also quantified the direct and indirect evidence contributing to each comparison.

## 2. Methods

### 2.1. Protocol and Reporting

This systematic review and network meta-analysis was reported in accordance with the Preferred Reporting Items for Systematic Reviews and Meta-Analyses (PRISMA) 2020 statement and the PRISMA extension for network meta-analyses (PRISMA-NMA) [19,20]. The review protocol was retrospectively registered with the Open Science Framework (OSF) on 16 August 2026 (registration DOI: 10.17605/OSF.IO/9MNDB). Registration occurred after preliminary searching, study selection, and exploratory analysis had begun.

### 2.2. Eligibility Criteria

Eligible participants were children and adolescents who received post-cardiac arrest care after return of spontaneous circulation (ROSC). We excluded neonatal resuscitation immediately after birth and neonatal hypoxic–ischemic encephalopathy. Studies defined by the original investigators as pediatric were eligible. Fink 2010 permitted enrollment up to 21 years but did not report the number aged ≥ 18 years; it contributed only to the expanded survival analysis [9]. We included randomized trials and comparative observational studies conducted after OHCA, IHCA, or both. Eligible interventions used a protocolized hypothermic temperature target with a maintenance duration that could be assigned to a 24, 48, or 72 h node. Comparators were another hypothermia duration, active normothermia, or usual care without protocolized induced hypothermia. We excluded case reports, single-arm series, adult-only studies, mixed or variable protocols that could not be assigned to a duration node and secondary reports of the same cohort from the primary NMA.

### 2.3. Information Sources and Search Strategy

We searched PubMed/MEDLINE, Embase, Scopus, Web of Science Core Collection, and the Cochrane Central Register of Controlled Trials (CENTRAL) from inception through 20 August 2026. We also searched ClinicalTrials.gov and checked the reference lists of the prior duration NMA and the recent pairwise review [15,16]. The main search combined controlled vocabulary and free-text terms for cardiac arrest or post-arrest care, pediatric populations, and temperature management concepts. We applied no restrictions on publication year, language, or document type and did not require a hypothermic duration term. Supplemental searches using duration and rewarming or maintenance terms were used to identify potential indexing or terminology gaps. The complete strategies and source-specific execution dates are provided in Supplementary S1.

### 2.4. Study Selection and Data Extraction

Retrieved records were managed and deduplicated using the EndNote 2025 reference management software (Clarivate, Philadelphia, PA, USA). Two reviewers (JK and TNC) independently screened titles and abstracts and assessed full-text eligibility. Disagreements were resolved through discussion and consensus. Using a standardized form, we extracted study design, country, arrest setting, eligibility criteria, sample size, arrest characteristics, target temperature, intended and actual duration, rewarming strategy, comparator temperature strategy, outcome definitions, assessment time points, and arm-level events and denominators. One reviewer extracted the data, and the other verified all entries against the source report. When survival or favorable outcome was derived as the complement of reported mortality or unfavorable outcome, the derivation was documented. Study authors were not contacted for additional data. Reported adverse events were extracted and summarized descriptively because their definitions and ascertainment windows differed.

### 2.5. Treatment Nodes and Comparator Definition

The network comprised hypothermia (HT) maintained for 24 h (HT-24 h), 48 h (HT-48 h), or 72 h (HT-72 h) and heterogeneous Control strategies (hereafter, Control). These durations refer to the intended hypothermic phase, not the total period of active temperature management. In the primary analyses, Control included active normothermia, standard temperature management with fever avoidance, and partly active normothermia. The expanded analyses additionally included usual care without a fixed temperature management protocol and no targeted temperature management. Thus, this comparison did not represent targeted temperature management (TTM) versus no TTM. Primary analyses required a fixed intended hypothermia duration and assignable arm-level outcomes; actual exposure could differ from the protocol-specified duration. A fixed intended-duration node required an explicitly stated 24, 48, or 72 h protocol; actual median exposure was not used to round a study into a node. A 36 h protocol would therefore not be assigned to either 24 or 48 h. Protocol deviations such as early death did not alter the intended assignment. Effect modifiers and study-level assignment rules are detailed in Supplementary Table S3.

Clinical transitivity was assessed by comparing arrest setting and etiology, age, resuscitation duration, time to target, rewarming, subsequent fever control, recruitment period, and outcome timing across direct comparisons. A restricted-network analysis retained only protocolized active-normothermia or direct-duration comparators; disconnected contrasts were reported as not estimable rather than combined across disconnected components.

### 2.6. Classification for Sensitivity Analyses and Overlapping Reports

Studies with otherwise-eligible comparative outcome data but approximate, pragmatic, or ranged durations that precluded unambiguous assignment to a fixed intended-duration node were excluded from the primary analysis and considered for a broader duration-classification sensitivity analysis. When two or more reports were derived from the same or partially overlapping cohort, they were not pooled simultaneously. We selected for the primary corpus the report that best matched the outcome definition and follow-up and then performed an overlapping report replacement sensitivity analysis using the alternative report. The Lin cohorts covered January 2010–June 2012 and January 2010–June 2017 [10,11]. The earlier report provided arm-level data for HT-72 h versus Control and was used as an alternative report in the replacement analysis. Secondary analyses of the same parent trial, such as the THAPCA timing report, did not provide an independent duration contrast and were excluded (Supplementary Table S4).

### 2.7. Outcome Definitions

The primary survival outcome was survival at the longest available time point of at least hospital discharge among fixed intended-duration studies. When a study reported multiple survival time points, we selected the longest according to the outcome hierarchy. The primary neurologic outcome was favorable neurologic survival at 6 months or later, with death classified as an unfavorable outcome. When validated scales differed, we retained the original study threshold for a favorable outcome and included deaths as unfavorable outcomes within the reported analysis population. THAPCA neurologic denominators were the evaluable baseline-eligible populations, not all randomized participants (138/122 for OHCA and 133/124 for IHCA); survival denominators reflected reported vital-status availability (151/136 and 166/161) [4,5]. The THAPCA trials used 12-month survival with a Vineland Adaptive Behavior Scales, second edition (VABS-II) score of at least 70; Lin et al. used a 6-month Pediatric Cerebral Performance Category (PCPC) score of 2 or less; and Fink et al. used PCPC at 6 months, defining unfavorable outcome as PCPC 4–6 or an increase of >1 from baseline [4,5,7,11]. Favorable Fink counts were derived as the complement of this definition. For Lin 2018 [11], the reported 7/15 and 1/12 favorable outcomes among 1-month survivors were expressed as 7/25 and 1/39 among all enrolled participants, with deaths classified as unfavorable. Arm-level counts and derivations are provided in Supplementary Table S5. Outcomes that did not meet the primary time-point criterion were designated exploratory when included in sensitivity analyses.

### 2.8. Risk-of-Bias Assessment

Risk of bias was assessed using the Cochrane Risk of Bias 2 (RoB 2) tool for randomized controlled trials and the Risk of Bias in Non-randomized Studies of Interventions (ROBINS-I) tool (original 2016 version) for observational studies [21,22]. JK and TNC performed the study-level risk-of-bias assessments, resolving disagreements by consensus. Outcome-specific judgments for survival and favorable neurologic outcome were based on source reports and available protocols and were reviewed and approved by TNC. Domain-level rationales are provided in Supplementary Table S10. Overall judgments from the two design-specific tools were presented separately rather than combined on a common scale.

### 2.9. Statistical Analysis

We calculated risk ratios (RRs) and 95% confidence intervals (CIs) from arm-level binary data. A frequentist random-effects NMA combined direct and indirect evidence under a common between-study heterogeneity variance for all treatment effects, estimated using restricted maximum likelihood (REML). Analyses were performed using R ver. 4.6.1 (R Foundation for Statistical Computing, Vienna, Austria) and the netmeta package ver. 3.6-1 [23]. Because every study in the networks contributed a two-arm comparison, no adjustment for within-study correlation arising from multi-arm trials was required. No zero-event cells occurred in the survival or favorable neurologic outcome contrasts included in the network analyses; therefore, continuity corrections were not applied. Statistical heterogeneity was summarized using τ^2^ and I^2^.

For each contrast, evidence-flow decomposition was used to calculate the proportion of the network estimate contributed by direct evidence [24]. Global inconsistency was assessed using design-by-treatment decomposition when estimable, and node splitting compared direct and indirect estimates for the three contrasts in the survival loop. Statistical incoherence could not be evaluated in the primary neurologic network because it contained no closed loop. Given the limited power of formal tests in sparse networks, clinical transitivity and network geometry were also considered. Treatment rankings and funnel plots were omitted because they could not support reliable inference. Certainty of evidence was assessed using Confidence in Network Meta-Analysis (CINeMA) across within-study bias, reporting bias, indirectness, imprecision, heterogeneity, and incoherence [25]. A clinical importance range of RR of 0.833–1.20, symmetric on the logarithmic scale, was selected post hoc for the certainty assessment. Overlapping concerns across domains were considered jointly rather than mechanically counted as independent downgrades. Reporting bias was assessed qualitatively in light of the small evidence base and registry information.

Two complementary sensitivity analyses addressed duration classification and overlapping report selection. The broader duration-classification sensitivity analysis relaxed the fixed intended-duration criterion and added approximate or pragmatic 24 h evidence. The overlapping report replacement sensitivity analysis substituted the primary report with an alternative report from the same study family. When a sensitivity analysis included a neurologic outcome with a timing or estimand that differed from the primary outcome at 6 months or later, it was interpreted as exploratory evidence. Namba et al. was assigned to HT-24 h only in the expanded analysis to test robustness to newer, predominantly hypothermia-based evidence. However, 26 of 91 temperature-managed patients received normothermia, and the 32–36 °C target range and approximately 24 h duration were not strictly standardized [17].

Additional post hoc analyses assessed omission of Lin 2018 [11], restriction to randomized trials, restriction to protocolized active-normothermia and direct-duration comparators, and survival follow-up of ≥6 months. A neurologic denominator sensitivity analysis used all THAPCA randomized participants while keeping observed favorable-event counts unchanged; all participants without a recorded favorable outcome, including those baseline-ineligible for the original outcome, were assumed unfavorable. This assumption-based analysis is not a reconstruction of a fully observed intention-to-treat outcome. Common-effect inverse-variance RR models and common-effect Mantel–Haenszel odds ratio (OR) models were examined as statistical robustness checks. The Mantel–Haenszel implementation in netmeta estimates ORs; these were compared with RR models only for direction and whether intervals included the null, not for numerical magnitude. Prediction intervals were calculated when residual degrees of freedom permitted; neurologic intervals were not estimable. Analysis-specific details are provided in Supplementary Table S7.

ChatGPT (OpenAI, San Francisco, CA, USA) was used as a reference during the preparation and refinement of R scripts; all statistical analyses were executed and independently verified by the authors. Further details regarding generative artificial intelligence (AI) use are provided in the Acknowledgments.

## 3. Results

### 3.1. Study Selection

The database searches identified 9357 records, and the ClinicalTrials.gov register search identified 72 records, yielding 9429 records in total. After 3385 duplicates were removed, 6044 records were screened, of which 5965 were excluded. All 79 reports sought for retrieval were obtained and assessed for eligibility; 71 were excluded with reasons (Supplementary Table S4). Reference list checking identified no additional eligible reports. Seven study families represented by eight reports contributed to the primary or sensitivity analyses. The primary survival NMA included five studies, and the primary neurologic NMA included four studies (Figure 1).

### 3.2. Study Characteristics

The eight reports comprised three randomized trial reports and five observational reports published from 2010 through 2025 [4,5,7,8,9,10,11,17]. The THAPCA-OH and THAPCA-IH studies were multicenter randomized trials comparing 48 h of hypothermia followed by active normothermia with active normothermia [4,5]. The pilot randomized trial by Fink et al. directly compared HT-24 h with HT-72 h in 34 participants already selected for hypothermia before randomization [7]. Scholefield et al. reported a protocolized 24 h cohort, explicitly described in the full-text temperature management protocol [8], whereas Lin et al. compared a 72 h protocol with partly active normothermia after asphyxial OHCA [11] (Table 1). Additional effect modifiers are provided in Supplementary Table S3.

### 3.3. Risk of Bias

In outcome-specific RoB 2 assessments, both THAPCA trials had low overall risk for survival but some concerns for neurologic outcome because caregiver input could influence VABS-II measurement despite blinded central interviewers [4,5]. Fink 2018 had some concerns for survival and high risk for neurologic outcome, particularly from outcome measurement [7]. All five observational reports retained an overall serious risk of bias for assessed outcomes, with serious confounding risk in every report [8,9,10,11,17]. Both Lin reports also had serious participant-selection risk [10,11]. Classification-of-intervention risk was moderate in Fink 2010 [9] and Namba 2025 [17] because duration or temperature exposure was not uniform; some domains in Namba had insufficient information. Detailed outcome-specific judgments and rationales are shown in Supplementary Figure S1 and Table S10.

### 3.4. Network Geometry

The primary survival network comprised four nodes and four types of direct comparison: HT-24 h versus Control, HT-48 h versus Control, HT-72 h versus Control, and HT-72 h versus HT-24 h (Figure 2A). The primary neurologic network had a tree structure comprising three direct comparisons: HT-48 h versus Control, HT-72 h versus Control, and HT-72 h versus HT-24 h (Figure 2B). Thus, the neurologic estimates for HT-24 h versus Control, HT-48 h versus HT-24 h, and HT-72 h versus HT-48 h were entirely indirect.

### 3.5. Primary Survival NMA

Compared with Control, the survival RR was 1.42 (95% CI, 0.83–2.44) for HT-24 h, 1.14 (95% CI, 0.94–1.37) for HT-48 h, and 2.01 (95% CI, 1.24–3.25) for HT-72 h. The RR was 1.41 (95% CI, 0.89–2.22) for HT-72 h versus HT-24 h, 1.77 (95% CI, 1.05–2.97) for HT-72 h versus HT-48 h, and 0.80 (95% CI, 0.45–1.41) for HT-48 h versus HT-24 h (Table 2; Figure 3A). Between-study heterogeneity was negligible (τ^2^ ≈ 0; I^2^ = 0%), and the design-by-treatment decomposition did not detect global inconsistency (between-design Q = 0.04, df = 1, *p* = 0.851); the single-loop test had little power and does not establish consistency.

The proportion of direct evidence was 51.3% for HT-24 h versus Control, 100% for HT-48 h versus Control, 72.0% for HT-72 h versus Control, and 76.7% for HT-72 h versus HT-24 h. No direct evidence contributed to HT-48 h versus HT-24 h or HT-72 h versus HT-48 h (Table 2; Supplementary Table S1). The 72.0% direct contribution to HT-72 h versus Control came from Lin 2018 [11] with 1-month survival, whereas the THAPCA contrast used 12-month survival. Direct, indirect, and network estimates for the survival loop are presented side by side in Supplementary Table S7.

### 3.6. Primary Favorable Neurologic Outcome NMA

Compared with Control, the RR for favorable neurologic outcome was 9.36 (95% CI, 0.89–98.38) for HT-24 h, 1.15 (95% CI, 0.69–1.92) for HT-48 h, and 10.92 (95% CI, 1.32–90.40) for HT-72 h. The RR was 1.17 (95% CI, 0.42–3.28) for HT-72 h versus HT-24 h, 9.50 (95% CI, 1.08–83.61) for HT-72 h versus HT-48 h, and 0.12 (95% CI, 0.01–1.36) for HT-48 h versus HT-24 h. Several contrasts, including HT-24 h and HT-72 h versus Control, had very wide CIs (Table 2; Figure 3B). The HT-72 h versus Control contrast depended entirely on Lin 2018 [11], with only one favorable event among 39 Control participants; its wide interval precludes a reliable estimate of effect magnitude.

The proportion of direct evidence was 100% for HT-48 h versus Control, HT-72 h versus Control, and HT-72 h versus HT-24 h. No direct evidence contributed to the remaining three contrasts (Table 2; Supplementary Table S2). Heterogeneity was moderate but highly uncertain (τ^2^ = 0.0857; I^2^ = 60.0%, 95% CI, 0.0–90.6%). The heterogeneity estimate was informed only by the repeated HT-48 h versus Control comparison in THAPCA-OH and THAPCA-IH (Q = 2.50, df = 1) and was applied to the single-study edges. Statistical incoherence could not be assessed because the neurologic network had a tree structure without a closed loop.

### 3.7. Sensitivity Analyses

The broader duration-classification survival analysis included 1273 participants from seven studies. The RR was 2.02 (95% CI, 1.22–3.34) for HT-72 h versus Control, 1.40 (95% CI, 0.85–2.31) for HT-72 h versus HT-24 h, 1.73 (95% CI, 0.94–3.18) for HT-72 h versus HT-48 h, and 1.44 (95% CI, 1.05–1.97) for HT-24 h versus Control. In the overlapping report replacement survival analysis, the corresponding RRs were 1.79 (95% CI, 1.16–2.74), 1.36 (95% CI, 0.87–2.13), and 1.57 (95% CI, 0.98–2.50) for HT-72 h versus Control, HT-24 h, and HT-48 h, respectively (Figure 4A). HT-24 h versus Control in the replacement survival analysis yielded an RR of 1.31 (95% CI, 0.78–2.20).

The broader duration-classification neurologic analysis included 922 participants from five studies. The RR was 4.38 (95% CI, 1.46–13.12) for HT-72 h versus Control, 1.45 (95% CI, 0.57–3.70) for HT-72 h versus HT-24 h, 3.81 (95% CI, 1.14–12.78) for HT-72 h versus HT-48 h, and 0.38 (95% CI, 0.16–0.92) for HT-48 h versus HT-24 h. In the overlapping report replacement neurologic analysis, the RR was 3.00 (95% CI, 1.11–8.09) for HT-72 h versus Control, 1.17 (95% CI, 0.42–3.28) for HT-72 h versus HT-24 h, and 2.61 (95% CI, 0.85–7.97) for HT-72 h versus HT-48 h (Figure 4B). HT-24 h versus Control in the replacement neurologic analysis yielded an RR of 2.57 (95% CI, 0.61–10.77).

The effect estimate for HT-72 h versus Control exceeded an RR of 1 in the primary, expanded, and replacement specifications. The CI for HT-72 h versus HT-24 h included an RR of 1 in these three specifications. Although the CIs for HT-72 h versus HT-48 h excluded 1 in the primary survival and neurologic analyses, they included 1 in the broader duration-classification survival analysis and in both replacement analyses (Figure 4A,B).

When Lin 2018 [11] was omitted, the survival estimates for HT-72 h versus Control and HT-48 h were 2.16 (95% CI, 0.86–5.43) and 1.90 (95% CI, 0.74–4.87), respectively; both intervals included no difference. The neurologic network became disconnected. Restricting either outcome to randomized trials or to protocolized active-normothermia and direct-duration comparators yielded two disconnected components: HT-48 h–Control and HT-24 h–HT-72 h. Consequently, HT-72 h versus HT-48 h or protocolized active-normothermia Control could not be estimated, whereas the direct HT-72 h versus HT-24 h comparison remained estimable. Treating the THAPCA comparator as a distinct Control node would likewise disconnect the 48 h component from the rest of the full network. Restricting survival to ≥6 months produced two components, with an RR of 1.14 (95% CI, 0.93–1.39) for HT-48 h versus Control and 1.44 (95% CI, 0.86–2.43) for HT-72 h versus HT-24 h (Supplementary Table S7).

Using all THAPCA randomized denominators under the unfavorable-outcome assumption yielded neurologic RRs of 10.92 (95% CI, 1.34–88.89) for HT-72 h versus Control and 9.18 (95% CI, 1.07–78.87) versus HT-48 h. This denominator change did not remove the extreme imprecision or the dependence on Lin 2018 [11] (Supplementary Table S7).

### 3.8. Statistical Robustness and Certainty of Evidence

Common-effect RR and Mantel–Haenszel OR models generally preserved the direction of the primary comparisons, but whether some neurologic confidence intervals included the null differed across models. For example, HT-24 h versus Control had a common-effect RR of 9.36 (95% CI, 1.03–85.15) compared with the random-effects interval of 0.89–98.38 and a Mantel–Haenszel OR of 11.52 (95% CI, 0.88–151.11). All survival prediction intervals crossed the null; neurologic prediction intervals were not estimable. Node splitting did not detect disagreement within the survival loop (*p* = 0.851 for each contrast) with limited power (Supplementary Table S7).

CINeMA confidence was moderate for survival with HT-48 h versus Control, low for survival with HT-72 h versus HT-24 h, and very low for the remaining four survival comparisons. All six neurologic comparisons had very low confidence. HT-72 h versus Control therefore had very low confidence for both outcomes (Supplementary Table S6). The neurologic tree did not permit empirical assessment of incoherence; major concerns were assigned according to CINeMA rules for networks without closed loops rather than because inconsistency had been demonstrated.

### 3.9. Safety and Ongoing Trials

Adverse event reporting was heterogeneous and did not permit a safety NMA. The THAPCA reports found no significant between-group differences in serious arrhythmias, culture-proven infections, or blood product use [4,5]. Fink 2018 [7] reported no significant adverse event differences between its small duration groups. Scholefield [8] reported more bradycardia and hypotension with hypothermia, and Lin 2018 [11] reported more bradycardia and coagulopathy; these observational comparisons cannot establish duration-specific harms. Arm-level results and ascertainment limitations are provided in Supplementary Table S8.

P-ICECAP (NCT05376267) was identified as an ongoing adaptive randomized trial of cooling duration after pediatric OHCA, with planned enrollment of up to 900 participants. The trial was listed as recruiting, with no published outcome results eligible for this synthesis (Supplementary Table S9) [18].

Under the primary fixed-duration criterion, the primary survival corpus included Scholefield et al., both THAPCA trials, the pilot trial by Fink et al., and the 2018 report by Lin et al. [4,5,7,8,11]. In the 2010 cohort reported by Fink et al., the median hypothermia duration was 24 h but the interquartile range was 16–48 h [9]. In the registry study by Namba et al., TTM at 32–36 °C was generally applied for approximately 24 h but was not implemented as a fixed-duration protocol [17]. We therefore classified these two studies as approximate or pragmatic 24 h evidence and included them only in the broader duration-classification sensitivity analysis. Only Namba et al., which reported a relevant neurologic outcome, was added to the neurologic sensitivity analysis.

The 2013 and 2018 reports by Lin et al. were not pooled simultaneously because the cohorts were recruited at the same institution and may have partially overlapped [10,11]. We selected the larger 2018 comparative cohort, which provided a 6-month outcome consistent with the primary neurologic outcome hierarchy, for the primary corpus [11]. The 2013 report replaced it in the overlapping report replacement sensitivity analysis [10].

## 4. Discussion

This NMA yielded three principal findings. First, HT-72 h showed favorable associations with survival and favorable neurologic outcome versus heterogeneous Control strategies, and the direction of effect was retained in the duration-classification and overlapping report replacement analyses. Second, superiority of HT-72 h over HT-24 h was not established in any analysis, including the comparison supported by direct randomized evidence. Third, the apparent advantage of HT-72 h over HT-48 h was entirely indirect and was not robust to study classification or overlapping report selection. However, omission of Lin 2018 [11] removed statistical significance for survival and disconnected the neurologic network, emphasizing study dependence rather than a stable duration effect. Certainty for the 72 h comparisons with Control was very low.

These findings should be interpreted within the current pediatric guideline framework. Active temperature management and fever prevention are recommended after pediatric cardiac arrest, and induction of hypothermia at 32–34 °C remains a guideline-supported option [1,2]. The THAPCA trials supplied the largest randomized evidence by evaluating a strategy that included 48 h of hypothermia followed by active normothermia [4,5]. Although their primary frequentist analyses did not show statistically significant superiority, the Bayesian reanalysis of THAPCA-OH estimated a high probability of some benefit from the hypothermia strategy [6]. Thus, induced hypothermia remains clinically plausible, but neither THAPCA nor the present network establishes the optimal duration of its hypothermic phase.

The favorable signal for HT-72 h versus Control was strongly influenced by observational evidence. The single-center study by Lin et al., involving children with asphyxial OHCA, contributed substantially to the primary survival estimate and supplied all direct evidence for the corresponding neurologic contrast [11]. Because treatment assignment was nonrandomized and the study had serious risk of bias from confounding and participant selection, baseline severity, resuscitation duration, treatment selection, and concurrent post-arrest care may have contributed to the observed association. Moreover, the Control node combined protocolized active normothermia, fever avoidance, partly active normothermia, and usual care without protocolized induced hypothermia. The Lin cohort excluded children dying within 12 h after ROSC, and exposure classification in this nonrandomized setting may introduce survivor selection or immortal time bias. The observational restriction to asphyxial OHCA further limits transportability. The contrast therefore cannot be interpreted as a pure causal effect of extending hypothermia to 72 h.

The duration comparisons provide a more restrained message. HT-72 h versus HT-24 h was informed by a direct pilot randomized trial, but that trial enrolled only 34 children and was not powered for definitive clinical outcomes [7]. All primary and sensitivity CIs included no difference; this indicates uncertainty, not equivalence. No study directly compared HT-72 h with HT-48 h. That estimate linked randomized 48 h evidence to observational 72 h evidence through different Control strategies and populations; restriction to protocolized active-normothermia and direct-duration comparators disconnected this comparison, and its statistical significance was lost in the expanded survival analysis and both overlapping report replacement analyses. Current evidence therefore does not establish that 72 h is superior to either 24 or 48 h.

The present study extends previous reviews by emphasizing robustness rather than treatment ranking. Pairwise reviews combined different hypothermia durations and therefore could not isolate a duration effect [14,15]. The prior NMA separated durations but pooled potentially overlapping reports and treated variable-duration evidence as fixed-duration evidence [16]. We restricted the primary corpus to fixed intended-duration studies, excluded simultaneous pooling of overlapping reports, aligned the minimum neurologic follow-up and accounted for deaths while retaining study-specific outcome definitions and analysis populations, quantified direct evidence contributions, and tested both classification and report selection in sensitivity analyses. Under this framework, the favorable direction of HT-72 h versus Control persisted, but superiority over shorter durations did not. This distinction is the principal methodological and clinical difference from the earlier NMA.

Clinical transitivity remained imperfect despite the common pediatric post-cardiac arrest setting. Studies differed in OHCA versus IHCA, arrest etiology, illness severity, resuscitation duration, comparator protocols, rewarming, fever prevention, and outcome definitions [3,4,5,7,8,9,10,11,17]. Survival timing ranged from discharge to 12 months, while favorable neurologic outcome was measured using different instruments, thresholds, and assessment procedures. VABS-II ≥ 70, PCPC ≤ 2, and the Fink definition incorporating change from baseline do not identify identical favorable-outcome populations. The primary outcome hierarchy reduced, but could not eliminate, this heterogeneity. Restriction to ≥6-month survival disconnected the network, underscoring the difficulty of bridging short- and long-term effects. The expanded analysis also included Namba et al., in which hypothermia predominated but 26 of 91 temperature-managed patients received normothermia and the intended exposure was not strictly standardized [17]. Its inclusion was useful as a stress test of the primary findings, but the resulting estimates remain limited as duration-specific evidence.

This review applied explicit duration-node rules, avoided simultaneous inclusion of potentially overlapping reports, and examined direct evidence proportions and complementary sensitivity analyses. Nevertheless, the network was small, combined randomized and observational designs, and depended heavily on single studies for several contrasts. All observational studies had serious risk of bias, neurologic estimates were highly imprecise, and the neurologic network contained no closed loop. The small number of studies also precluded meaningful subgroup analysis, meta-regression, reliable evaluation of publication bias, and formal evaluation of neurologic incoherence. CINeMA identified very low confidence for most comparisons, although survival with HT-48 h versus Control had moderate confidence. Retrospective registration leaves uncertainty about analytic choices made after preliminary results were known. Study authors were not contacted, and registry searching was limited to ClinicalTrials.gov. Safety data were sparse and inconsistently ascertained. These limitations constrain causal interpretation; differences in statistical specification cannot overcome intransitivity or observational bias.

Clinically, the results indicate persistent equipoise rather than support for routine 72 h hypothermia. They do not challenge guideline-based active temperature control or the option of an initial hypothermic phase, but they provide no basis for changing current practice to a uniform longer duration. Adequately powered trials should directly compare hypothermia durations while standardizing target temperature, induction, rewarming, subsequent fever prevention, and major cointerventions. Long-term neurologic outcomes should be prespecified using validated scales and denominators that include deaths. The lack of a demonstrated advantage for longer cooling in adult cardiac arrest and neonatal encephalopathy also cautions against extrapolating a “longer is better” principle [12,13]. P-ICECAP is designed to provide more informative direct pediatric duration evidence [18].

## 5. Conclusions

In pediatric post-cardiac arrest care, favorable associations for HT-72 h versus heterogeneous Control strategies depended predominantly on a single observational study at serious risk of bias, with very low certainty for both outcomes. However, sensitivity analyses did not establish robust superiority over HT-24 h, and the apparent advantage over HT-48 h was entirely indirect and specification-dependent. The available network therefore does not identify an optimal hypothermia duration or support changing current practice. Adequately powered direct comparative trials using standardized temperature protocols and long-term neurologic outcomes are needed; the ongoing P-ICECAP trial directly addresses this question.

## Figures and Tables

**Figure 1:**
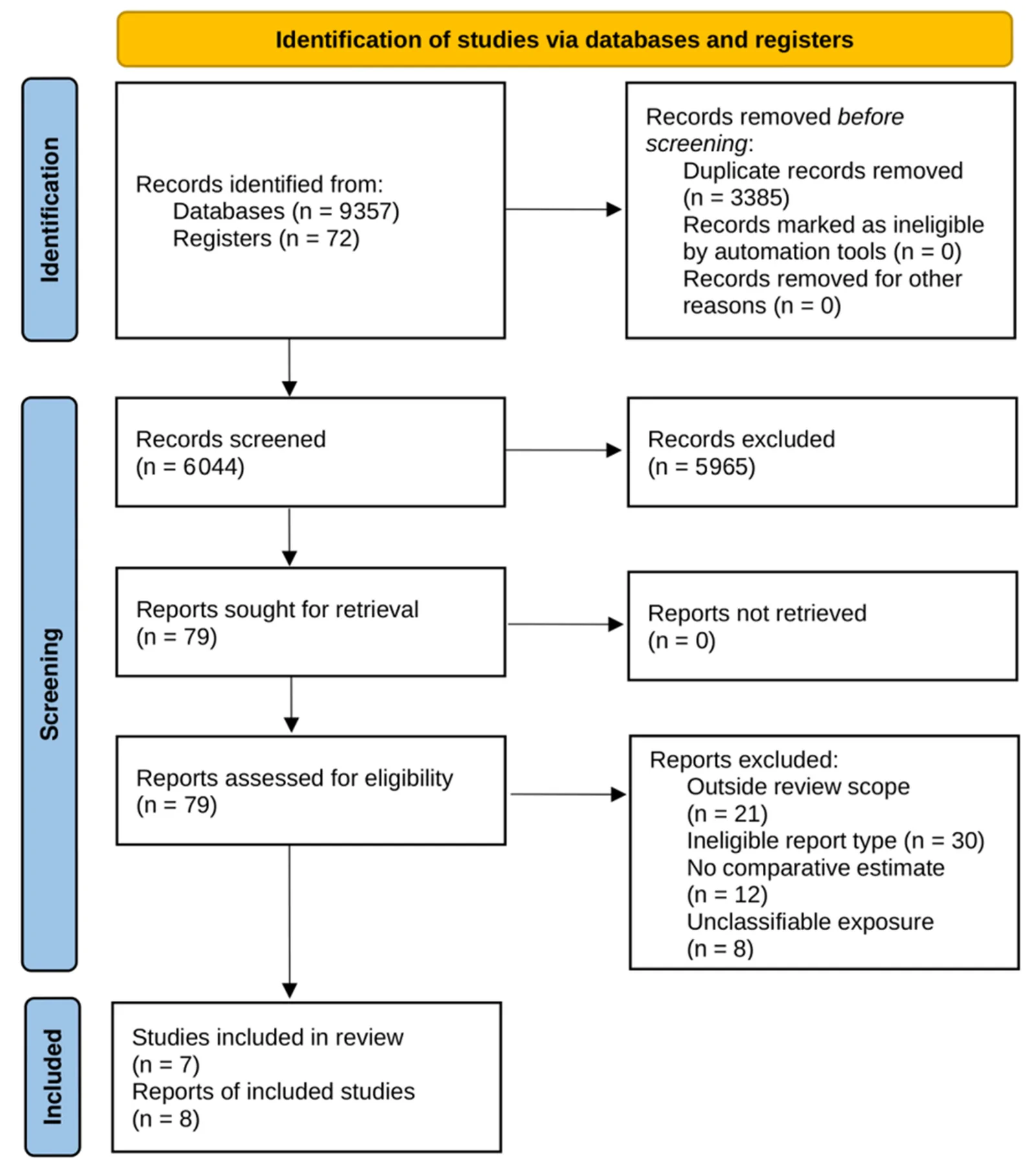
Preferred Reporting Items for Systematic Reviews and Meta-Analyses 2020 flow diagram of study selection. The diagram summarizes records identified through database and register searches, screening and full-text assessment, reasons for exclusion, and reports included in the analyses. Report-level exclusion reasons are listed in Supplementary Table S4. Reference list screening identified no additional eligible reports. Primary survival and neurologic syntheses included five and four studies, respectively; broader sensitivity syntheses included seven and five. Eight reports representing seven study families contributed to the primary or sensitivity analyses. The two potentially overlapping Lin reports [10,11] were never pooled in the same network. Adapted from the PRISMA 2020 flow diagram (Page et al. [19]).

**Figure 2:**
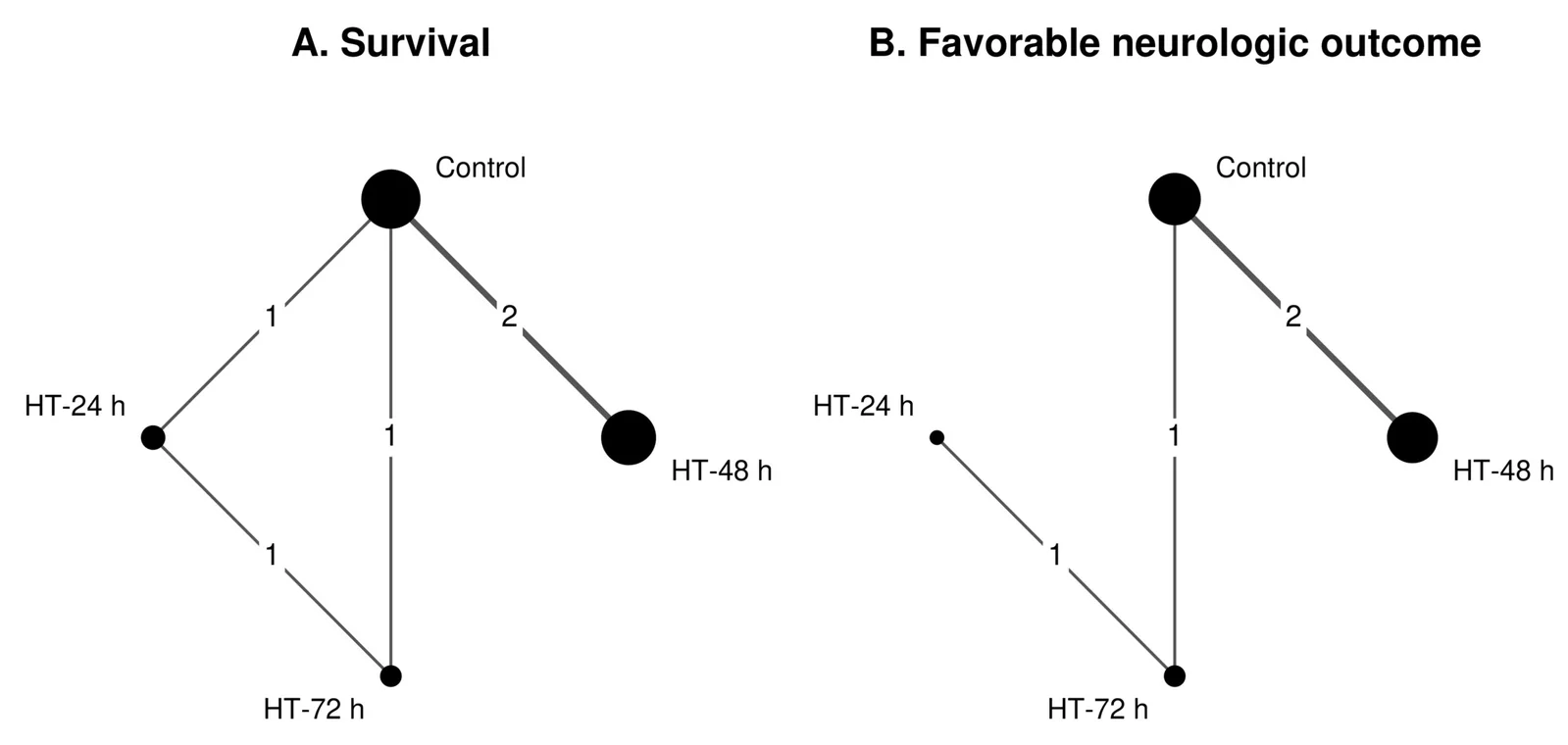
Network geometry of the primary analyses. (**A**) Survival. (**B**) Favorable neurologic outcome. Nodes represent treatment strategies, with area proportional to the number of participants analyzed for the respective outcome. Edges indicate direct comparisons, their thickness distinguishes the amount of direct study evidence, and edge labels give the number of studies. The neurologic network has no closed loop. Control denotes heterogeneous comparator strategies, including active normothermia, standard temperature management or fever avoidance, and partly active normothermia. h, hours; HT, hypothermia.

**Figure 3:**
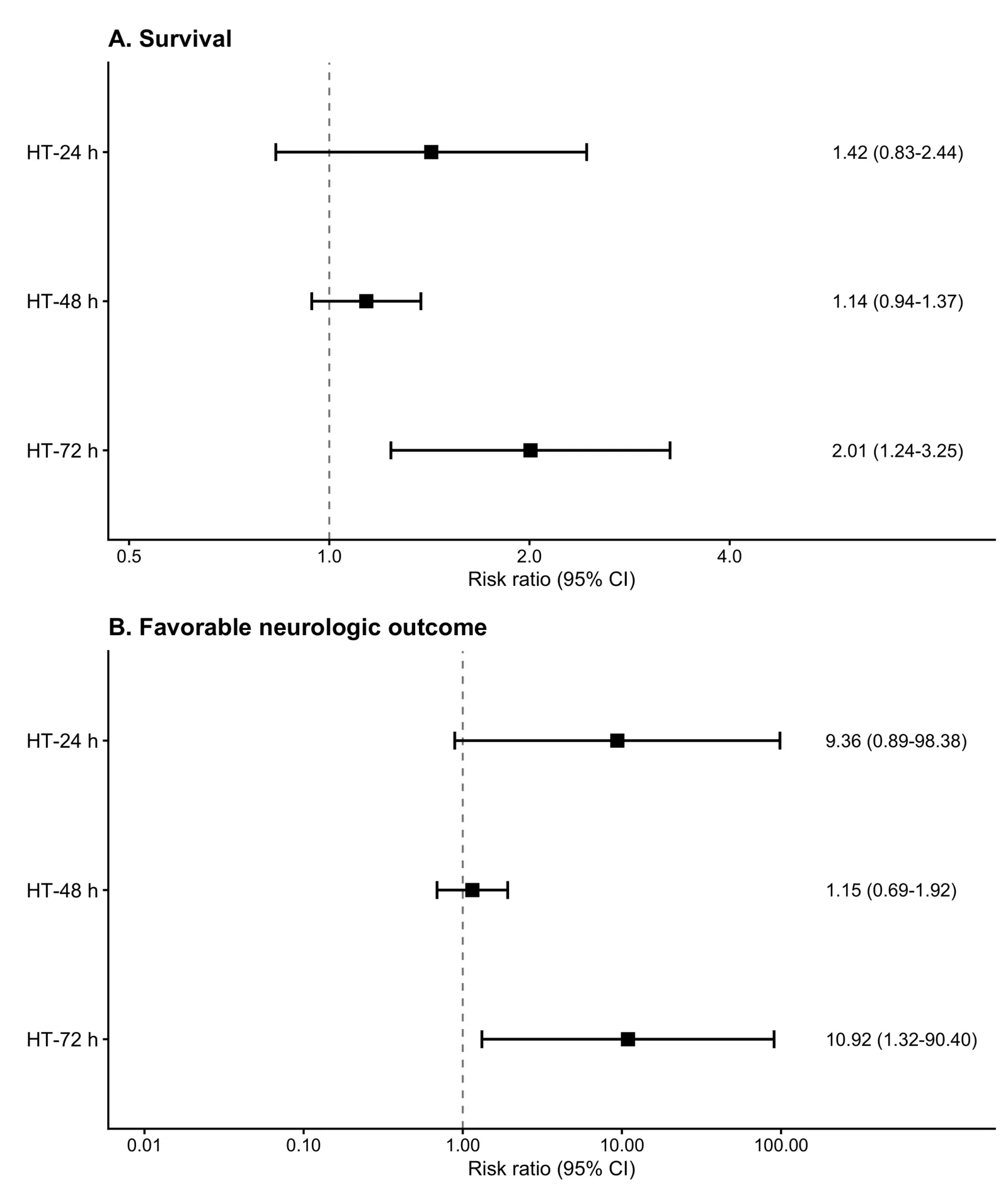
Primary network meta-analysis estimates versus Control. (**A**) Survival. (**B**) Favorable neurologic outcome. Points and horizontal lines show random-effects network risk ratios and 95% confidence intervals. The vertical reference line indicates no difference; risk ratios > 1 favor the hypothermia strategy. Horizontal axes use logarithmic scales and differ between panels. Control denotes heterogeneous comparator strategies, including active normothermia, standard temperature management or fever avoidance, and partly active normothermia. CI, confidence interval; h, hours; HT, hypothermia.

**Figure 4:**
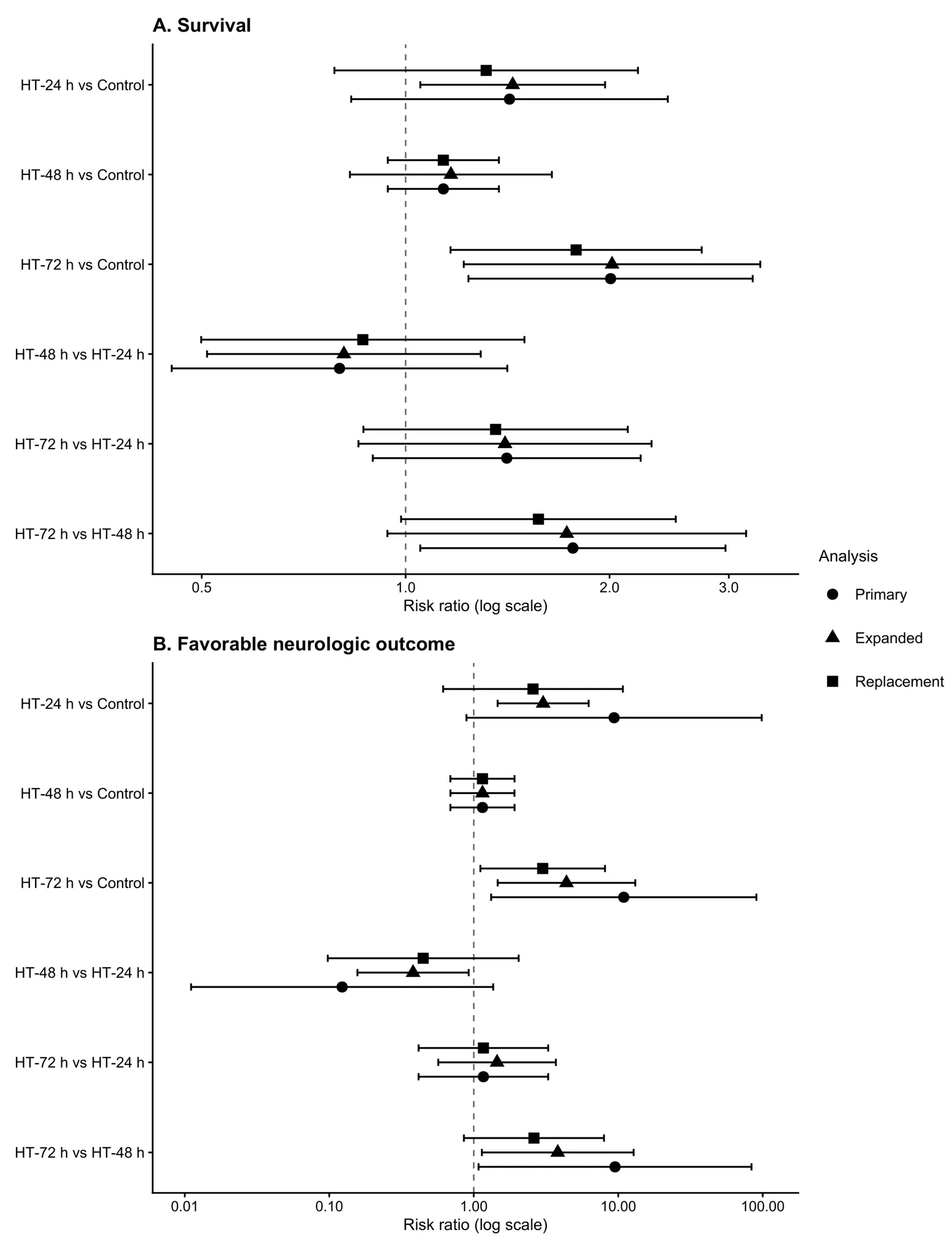
Sensitivity analyses of duration-specific comparisons. (**A**) Survival. (**B**) Favorable neurologic outcome. Points and horizontal lines show network risk ratios and 95% confidence intervals. Primary denotes fixed intended-duration evidence; Expanded adds approximate or pragmatic 24 h evidence; Replacement substitutes Lin 2013 [10] for Lin 2018 [11] without pooling the two reports. Expanded and replacement neurologic analyses include shorter-term outcomes and are exploratory. Risk ratios > 1 favor the treatment named first. Axes differ between panels. h, hours; HT, hypothermia. CI, confidence interval.

**Table 1:** Characteristics of included study reports.

Study	Design and Setting	Population	N	Hypothermia Strategy	Comparator	Outcomes Used	NMA Role
Fink 2010 [9]	Retrospective observational; single-center; USA; IHCA/OHCA	Age 1 wk–21 y; ROSC; complex congenital heart disease excluded	181 (40/141)	Median 34.0 °C; median 24 h (IQR 16–48 h); approximate HT-24 h	Usual care/no fixed active temperature protocol	Hospital survival	Expanded survival
Lin 2013 [10]	Retrospective observational; single-center; Taiwan; IHCA/OHCA	Age 2 mo–18 y; CPR ≥ 3 min; survival ≥ 12 h after ROSC; asphyxial comparative cohort	42 comparative (14/28)	33 ± 0.5 °C for 72 h; HT-72 h	Normothermia/fever prevention; active warming if needed	Hospital survival; favorable neurologic outcome at discharge	Replacement sensitivity
Scholefield 2015 [8]	Retrospective observational; single-center; UK; OHCA	Age > 1 d–16 y; admitted to PICU after OHCA	73 (38/35)	32–34 °C for 24 h; actual median 22.5 h; HT-24 h	Standard temperature management aiming < 38 °C	Hospital survival	Primary survival
THAPCA-OH 2015 [4]	Multicenter RCT; USA/Canada/UK; OHCA	Age > 2 d and <18 y; comatose after ROSC	295 randomized (155/140)	33 °C (32–34 °C) for 48 h; active temperature management continued to 120 h; HT-48 h	Active normothermia at 36.8 °C (36–37.5 °C) for 120 h	12 mo survival; favorable neurobehavioral survival at 12 mo	Primary survival + neurologic
THAPCA-IH 2017 [5]	Multicenter RCT; USA/Canada/UK; IHCA	Age > 48 h and <18 y; comatose after ROSC	329 randomized (166/163)	33 °C (32–34 °C) for 48 h; active temperature management continued to 120 h; HT-48 h	Active normothermia at 36.8 °C (36–37.5 °C) for 120 h	12 mo survival; favorable neurobehavioral survival at 12 mo	Primary survival + neurologic
Fink 2018 [7]	Pilot RCT; single-center; USA; IHCA/OHCA	Age 1 wk–17 y; comatose after ROSC; hypothermia initiated before randomization	34 (17/17)	33 ± 1 °C for 24 vs. 72 h; actual target exposure ~30 vs. ~78 h	HT-24 h vs. HT-72 h (direct duration comparison)	6 mo survival; favorable neurologic outcome at 6 mo	Primary survival + neurologic
Lin 2018 [11]	Retrospective observational; single-center; Taiwan; asphyxial OHCA	Age 1 mo–18 y; asphyxial OHCA; survival ≥12 h after ROSC	64 (25/39)	33 ± 0.5 °C for 72 h; HT-72 h	Normothermia 35.5–37.5 °C; partly active temperature control	1 mo survival; favorable neurologic outcome at 6 mo	Primary survival + neurologic
Namba 2025 [17]	Multicenter observational registry; Japan; OHCA	Age ≤ 18 y; OHCA with ROSC	307 (91/216)	32–36 °C, typically 24 h; pragmatic approximate HT-24 h	No targeted temperature management	30 d survival; favorable neurologic outcome at 30 d	Expanded survival + neurologic

Sample sizes describe the source cohorts; analysis-specific denominators may differ by outcome. Parentheses show intervention/comparator group sizes (24 h/72 h for Fink 2018 [7]). HT-24 h, HT-48 h, and HT-72 h denote the hypothermia duration nodes. Approximate-duration evidence was used only in expanded analyses; the Lin reports were never pooled together. CPR, cardiopulmonary resuscitation; d, days; h, hours; HT, hypothermia; IHCA, in-hospital cardiac arrest; IQR, interquartile range; min, minutes; mo, months; N, number of participants; NMA, network meta-analysis; OHCA, out-of-hospital cardiac arrest; PICU, pediatric intensive care unit; RCT, randomized controlled trial; ROSC, return of spontaneous circulation; THAPCA-IH, Therapeutic Hypothermia After Pediatric Cardiac Arrest—in-hospital trial; THAPCA-OH, Therapeutic Hypothermia After Pediatric Cardiac Arrest—out-of-hospital trial; UK, United Kingdom; USA, United States of America; wk, weeks; y, years.

**Table 2:** Primary network meta-analysis estimates and direct-evidence proportions.

Comparison	Survival, RR (95% CI)	Direct Evidence, %	Favorable Neurologic Outcome, RR (95% CI)	Direct Evidence, %
HT-24 h vs. Control	1.42 (0.83–2.44)	51.3	9.36 (0.89–98.38)	0
HT-48 h vs. Control	1.14 (0.94–1.37)	100	1.15 (0.69–1.92)	100
HT-72 h vs. Control	2.01 (1.24–3.25)	72	10.92 (1.32–90.40)	100
HT-48 h vs. HT-24 h	0.80 (0.45–1.41)	0	0.12 (0.01–1.36)	0
HT-72 h vs. HT-24 h	1.41 (0.89–2.22)	76.7	1.17 (0.42–3.28)	100
HT-72 h vs. HT-48 h	1.77 (1.05–2.97)	0	9.50 (1.08–83.61)	0

RR > 1 favors the treatment named first. Direct-evidence proportions were obtained using evidence flow decomposition. Control includes active normothermia and other comparator strategies without protocolized induced hypothermia. CI, confidence interval; h, hours; HT, hypothermia; NMA, network meta-analysis; RR, risk ratio.

## Data Availability

The data analyzed in this study were extracted from previously published studies cited in this article. The extracted study-level dataset and R analysis scripts are available from the corresponding author upon reasonable request.

## Supplementary Materials

The following supporting information can be downloaded at: https://www.mdpi.com/article/10.3390/jcm15218364/s1. Table S1: Primary survival league table; Table S2: Primary favorable neurologic outcome league table; Figure S1: Outcome-specific risk-of-bias assessments; Table S3: Treatment assignment and clinical transitivity; Table S4: All 71 excluded reports and reasons; Table S5A: Arm-level survival data and individual-study estimates; Table S5B: Arm-level neurologic outcome data and individual-study estimates; Table S6: CINeMA certainty assessment—(A) Survival and (B) Favorable neurologic outcome; Table S7A: Influence, restriction, and denominator analyses; Table S7B: Common-effect versus random-effects RR models; Table S7C: Mantel–Haenszel common-effect OR models; Table S7D: Random-effects prediction intervals; Table S7E: Direct, indirect, and network estimates in the survival loop; Table S7F: THAPCA neurologic populations and denominator sensitivity; Table S8: Reported adverse events and safety outcomes; Table S9: Ongoing randomized duration trial; Table S10A: Outcome-specific RoB 2 judgments; Table S10B: Outcome-specific ROBINS-I judgments; Table S10C: Principal domain-level rationales; Supplementary S1: Search strategies.

## References

[1] Lasa J.J., Dhillon G.S., Duff J.P., Hayes J., Kamath-Rayne B.D., Levy A., Mahgoub M., Morgan R.W., McCormick T., Roberts J.S. (2025). Part 8: Pediatric advanced life support: 2025 American Heart Association and American Academy of Pediatrics Guidelines for Cardiopulmonary Resuscitation and Emergency Cardiovascular Care. Circulation.

[2] Kim D.K., Kim J.T., Na J.Y., Park B., Lee J., Jeong S.I., Park J.D., Chung S.P., Kim T.-Y., Sohn Y. (2026). 2025 Korean Guidelines for Cardiopulmonary Resuscitation. Part 8. Pediatric advanced life support. Clin. Exp. Emerg. Med..

[3] Topjian A.A., de Caen A., Wainwright M.S., Abella B.S., Abend N.S., Atkins D.L., Bembea M.M., Fink E.L., Guerguerian A.-M., Haskell S.E. (2019). Pediatric post-cardiac arrest care: A scientific statement from the American Heart Association. Circulation.

[4] Moler F.W., Silverstein F.S., Holubkov R., Slomine B.S., Christensen J.R., Nadkarni V.M., Meert K.L., Clark A.E., Browning B., Pemberton V.L. (2015). Therapeutic hypothermia after out-of-hospital cardiac arrest in children. N. Engl. J. Med..

[5] Moler F.W., Silverstein F.S., Holubkov R., Slomine B.S., Christensen J.R., Nadkarni V.M., Meert K.L., Browning B., Pemberton V.L., Page K. (2017). Therapeutic hypothermia after in-hospital cardiac arrest in children. N. Engl. J. Med..

[6] Harhay M.O., Blette B.S., Granholm A., Moler F.W., Zampieri F.G., Goligher E.C., Gardner M.M., Topjian A.A., Yehya N. (2023). A Bayesian interpretation of a pediatric cardiac arrest trial (THAPCA-OH). NEJM Evid..

[7] Fink E.L., Clark R.S.B., Berger R.P., Fabio A., Angus D.C., Watson R.S., Gianakas J.J., Panigrahy A., Callaway C.W., Bell M.J. (2018). 24 vs. 72 hours of hypothermia for pediatric cardiac arrest: A pilot, randomized controlled trial. Resuscitation.

[8] Scholefield B.R., Morris K.P., Duncan H.P., Perkins G.D., Gosney J., Skone R., Sanders V., Gao F. (2015). Evolution, safety and efficacy of targeted temperature management after pediatric cardiac arrest. Resuscitation.

[9] Fink E.L., Clark R.S.B., Kochanek P.M., Bell M.J., Watson R.S. (2010). A tertiary care center’s experience with therapeutic hypothermia after pediatric cardiac arrest. Pediatr. Crit. Care Med..

[10] Lin J.J., Hsia S.H., Wang H.S., Chiang M.C., Lin K.L. (2013). Therapeutic hypothermia associated with increased survival after resuscitation in children. Pediatr. Neurol..

[11] Lin J.J., Lin C.Y., Hsia S.H., Wang H.S., Chiang M.C., Lin K.L., iCNS Group (2018). 72-h therapeutic hypothermia improves neurological outcomes in paediatric asphyxial out-of-hospital cardiac arrest—An exploratory investigation. Resuscitation.

[12] Shankaran S., Laptook A.R., Pappas A., McDonald S.A., Das A., Tyson J.E., Poindexter B.B., Schibler K., Bell E.F., Heyne R.J. (2014). Effect of depth and duration of cooling on deaths in the NICU among neonates with hypoxic ischemic encephalopathy: A randomized clinical trial. JAMA.

[13] Kirkegaard H., Søreide E., de Haas I., Pettilä V., Taccone F.S., Arus U., Storm C., Hassager C., Nielsen J.F., Sørensen C.A. (2017). Targeted temperature management for 48 vs. 24 hours and neurologic outcome after out-of-hospital cardiac arrest: A randomized clinical trial. JAMA.

[14] Buick J.E., Wallner C., Aickin R., Meaney P.A., de Caen A., Maconochie I.K., Skrifvars M.B., Welsford M., Meaney P., Atkins D. (2019). Paediatric targeted temperature management post cardiac arrest: A systematic review and meta-analysis. Resuscitation.

[15] Alsabri M., Rath S., Kamal I., Abdelrahman S.T., Aziz M.M., Lusinski E., Saleh Z. (2026). Targeted temperature management strategies in pediatric patients with return of spontaneous circulation after out-of-hospital cardiac arrest: A Grading of Recommendations, Assessment, Development, and Evaluation-assessed systematic review and meta-analysis. Ther. Hypothermia Temp. Manag..

[16] Amagasa S., Yasuda H., Oishi T., Kodama S., Kashiura M., Moriya T. (2022). Target temperature management following pediatric cardiac arrest: A systematic review and network meta-analysis to compare the effectiveness of the length of therapeutic hypothermia. Cureus.

[17] Namba T., Nishikimi M., Emoto R., Kikutani K., Ohshimo S., Matsui S., Shime N. (2025). Effect size of targeted temperature management in pediatric patients with post-cardiac arrest syndrome according to the severity. Life.

[18] ClinicalTrials.gov Pediatric Influence of Cooling Duration on Efficacy in Cardiac Arrest Patients (P-ICECAP). NCT05376267. NCT05376267.

[19] Page M.J., McKenzie J.E., Bossuyt P.M., Boutron I., Hoffmann T.C., Mulrow C.D., Shamseer L., Tetzlaff J.M., Akl E.A., Brennan S.E. (2021). The PRISMA 2020 statement: An updated guideline for reporting systematic reviews. BMJ.

[20] Hutton B., Salanti G., Caldwell D.M., Chaimani A., Schmid C.H., Cameron C., Ioannidis J.P.A., Straus S., Thorlund K., Jansen J.P. (2015). The PRISMA extension statement for reporting of systematic reviews incorporating network meta-analyses of health care interventions: Checklist and explanations. Ann. Intern. Med..

[21] Sterne J.A.C., Savović J., Page M.J., Elbers R.G., Blencowe N.S., Boutron I., Cates C.J., Cheng H.Y., Corbett M.S., Eldridge S.M. (2019). RoB 2: A Revised Tool for Assessing Risk of Bias in Randomised Trials.

[22] Sterne J.A., Hernán M.A., Reeves B.C., Savović J., Berkman N.D., Viswanathan M., Henry D., Altman D.G., Ansari M.T., Boutron I. (2016). ROBINS-I: A tool for assessing risk of bias in non-randomised studies of interventions. BMJ.

[23] Balduzzi S., Rücker G., Nikolakopoulou A., Papakonstantinou T., Salanti G., Efthimiou O., Schwarzer G. (2023). netmeta: An R package for network meta-analysis using frequentist methods. J. Stat. Softw..

[24] König J., Krahn U., Binder H. (2013). Visualizing the flow of evidence in network meta-analysis and characterizing mixed treatment comparisons. Stat. Med..

[25] Nikolakopoulou A., Higgins J.P.T., Papakonstantinou T., Chaimani A., Del Giovane C., Egger M., Salanti G. (2020). CINeMA: An approach for assessing confidence in the results of a network meta-analysis. PLoS Med..

