# Supplementary material for: Duration of Induced Hypothermia After Pediatric Cardiac Arrest: A Systematic Review and Network Meta-Analysis
Source: J Clin Med. 2026 Oct 27;15(21):8364. doi: 10.3390/jcm15218364 (PMC13764530; doi:10.3390/jcm15218364)
Supplement: Supplementary file 1 [file jcm-15-08364-s001.zip › jcm-4567742-supplementary.pdf]

Supplementary Material

Duration of Induced Hypothermia after Pediatric Cardiac Arrest: A Systematic Review and Network Meta-Analysis

Jinpyo Kim and Tae Nyoung Chung

Supplementary Table S1. Primary survival league table

Row treatment / column treatment	Control	HT-24 h	HT-48 h	HT-72 h
Control	—			
HT-24 h	1.42 (0.83–2.44)	—		
HT-48 h	1.14 (0.94–1.37)	0.80 (0.45–1.41)	—	
HT-72 h	2.01 (1.24–3.25)	1.41 (0.89–2.22)	1.77 (1.05–2.97)	—

Values are risk ratios (95% confidence intervals) from random-effects network meta-analysis. Each lower-triangle cell compares the row treatment with the column treatment; a risk ratio greater than 1 favors the row treatment. The upper triangle is omitted to avoid duplicate comparisons, and dashes indicate self-comparisons.

The primary survival analysis included five studies and 785 participants and used the longest available survival follow-up of at least hospital discharge. Control denotes heterogeneous comparator strategies. HT, hypothermia; h, hours.

Supplementary Table S2. Primary favorable neurologic outcome league table

Row treatment / column treatment	Control	HT-24 h	HT-48 h	HT-72 h
Control	—			
HT-24 h	9.36 (0.89–98.38)	—		
HT-48 h	1.15 (0.69–1.92)	0.12 (0.01–1.36)	—	
HT-72 h	10.92 (1.32–90.40)	1.17 (0.42–3.28)	9.50 (1.08–83.61)	—

Values are risk ratios (95% confidence intervals) from random-effects network meta-analysis. Each lower-triangle cell compares the row treatment with the column treatment; a risk ratio greater than 1 favors the row treatment. The upper triangle is omitted to avoid duplicate comparisons, and dashes indicate self-comparisons.

The primary neurologic analysis included four studies and 615 participants and evaluated favorable neurologic survival at 6 months or later, with deaths classified as unfavorable outcomes.

Control denotes heterogeneous comparator strategies. CI, confidence interval; h, hours; HT, hypothermia; RR, risk ratio.

Supplementary Figure S1. Outcome-specific risk-of-bias assessments

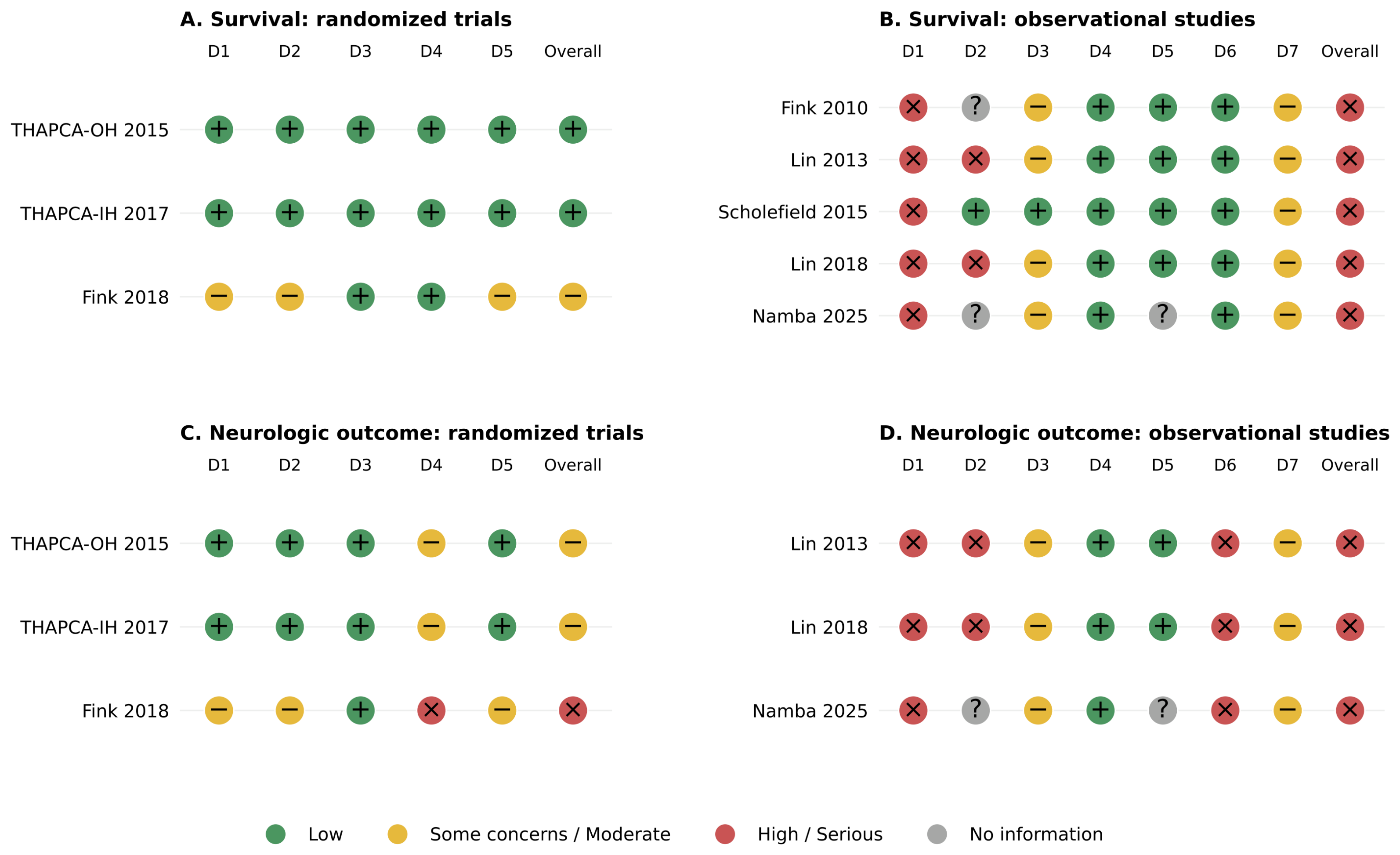

Outcome-specific risk-of-bias assessments. Panels A and C show randomized trials assessed using RoB 2; panels B and D show observational studies assessed using ROBINS-I. A/B: survival. C/D: favorable neurologic outcome. Domain definitions are provided in Supplementary Table S10. Green (+), low; yellow (−), some concerns for RoB 2 or moderate for ROBINS-I; red (×), high for RoB 2 or serious for ROBINS-I; gray (?), no information. Ratings are outcome specific. The figure includes studies contributing to primary or sensitivity analyses; it does not imply that overlapping Lin cohorts were pooled together.

Supplementary Table S3. Treatment assignment and clinical transitivity

A fixed node requires an explicit intended 24-, 48-, or 72-hour protocol. Actual exposure need not equal intended duration. Approximate medians, variable individual durations, and mixed normothermic exposure are not treated as fixed protocols. Data below describe source cohorts and are potential effect modifiers, not adjusted covariates.

Study / analysis role	Design, setting and eligibility	Intended duration / classification	Comparator and follow-up
Fink 2010; Expanded survival	Retrospective observational; single-center; USA; IHCA/OHCA; Age 1 wk–21 y; ROSC; complex congenital heart disease excluded	Median 34.0°C; median 24 h (IQR 16–48 h); approximate HT-24 h	Usual care / no fixed active temperature protocol; Hospital survival
Lin 2013; Replacement sensitivity	Retrospective observational; single-center; Taiwan; IHCA/OHCA; Age 2 mo–18 y; CPR ≥3 min; survival ≥12 h after ROSC; asphyxial comparative cohort	33 ± 0.5°C for 72 h; HT-72 h	Normothermia / fever prevention; active warming if needed; Hospital survival; favorable neurologic outcome at discharge
Scholefield 2015; Primary survival	Retrospective observational; single-center; UK; OHCA; Age >1 d–16 y; admitted to PICU after OHCA	32–34°C for 24 h; actual median 22.5 h; HT-24 h	Standard temperature management aiming <38°C; Hospital survival
THAPCA-OH 2015; Primary survival + neurologic	Multicenter RCT; USA/Canada/UK; OHCA; Age >2 d and <18 y; comatose after ROSC	33°C (32–34°C) for 48 h; active temperature management continued to 120 h; HT-48 h	Active normothermia at 36.8°C (36–37.5°C) for 120 h; 12-mo survival; favorable neurobehavioral survival at 12 mo
THAPCA-IH 2017; Primary survival + neurologic	Multicenter RCT; USA/Canada/UK; IHCA; Age >48 h and <18 y; comatose after ROSC	33°C (32–34°C) for 48 h; active temperature management continued to 120 h; HT-48 h	Active normothermia at 36.8°C (36–37.5°C) for 120 h; 12-mo survival; favorable neurobehavioral survival at 12 mo
Fink 2018; Primary survival + neurologic	Pilot RCT; single-center; USA; IHCA/OHCA; Age 1 wk–17 y; comatose after ROSC; hypothermia initiated before randomization	33 ± 1°C for 24 vs 72 h; actual target exposure ~30 vs ~78 h	HT-24 h vs HT-72 h (direct duration comparison); 6-mo survival; favorable neurologic outcome at 6 mo
Lin 2018; Primary survival + neurologic	Retrospective observational; single-center; Taiwan; asphyxial OHCA; Age 1 mo–18 y; asphyxial OHCA; survival ≥12 h after ROSC	33 ± 0.5°C for 72 h; HT-72 h	Normothermia 35.5–37.5°C; partly active temperature control; 1-mo survival; favorable neurologic outcome at 6 mo
Namba 2025; Expanded survival + neurologic	Multicenter observational registry; Japan; OHCA; Age ≤18 y; OHCA with ROSC	32–36°C, typically 24 h; pragmatic approximate HT-24 h	No targeted temperature management; 30-d survival; favorable neurologic outcome at 30 d

Supplementary Table S3 continued. Potential effect modifiers across primary comparisons

Study / direct comparison	Recruitment / setting	Age, etiology and severity	Resuscitation duration	Cooling and rewarming	Subsequent control / outcomes
Scholefield 2015; HT-24 h vs Control	2004–2010; OHCA	Median age 1.5 (IQR 0–5.8) vs 1.0 (0–4.0) y; mixed etiology; pulmonary 24% vs 20%	Arrest-to-ROSC median 40 (26–56) vs 29 (21–46) min	Cooling-start-to-target median 2 h (IQR 0–3 h 15 min); actual target exposure median 22.5 h; rewarming median 0.3°C/h	Standard management <38°C; hospital survival
THAPCA-OH 2015; HT-48 h vs Control	September 2009–December 2012; OHCA	Median age 2.1 (0.6–10.1) vs 1.6 (0.4–7.0) y; respiratory etiology 72% vs 73%	CPR median 23 (15–35) vs 28 (19–45) min, available in 186 participants	Randomized within 6 h of ROSC; ROSC-to-treatment initiation median 5.9 (5.2–6.7) vs 5.8 (5.0–6.4) h; time to target not reported in the primary report; rewarming ≥16 h	36.8°C (36–37.5°C) to 120 h in both strategies; survival and VABS-II at 12 months
THAPCA-IH 2017; HT-48 h vs Control	September 2009–February 2015; IHCA	Median age 1.4 (0.3–5.7) vs 0.6 (0.2–6.3) y; cardiac/congenital-heart etiology ~65% overall; ECMO before randomization 52% vs 58%	CPR median 23 (7–42) vs 22 (7–51) min, available in 321 participants	Randomized within 6 h; ROSC-to-treatment initiation median 4.9 (3.9–5.8) vs 4.7 (4.0–5.7) h; ROSC-to-target ~6 h; rewarming ≥16 h	36.8°C (36–37.5°C) to 120 h in both strategies; survival and VABS-II at 12 months
Fink 2018; HT-24 h vs HT-72 h	November 2009–December 2013; 82% OHCA	Eligible 1 week–17 years; asphyxial etiology 94% vs 82%	CPR-to-ROSC median 17 (10–27.5) vs 25.5 (17.5–30) min	HT initiated before randomization; post-randomization time to target overall median 0 (0–2) h; rewarming 0.5°C every 4 h	Rewarmed to 37°C; duration of subsequent fever control NR; survival and PCPC at 6 months
Lin 2018; HT-72 h vs Control	January 2010–June 2017; asphyxial OHCA	All arrests asphyxial; age <1 y in 52% vs 33.3%; survival ≥12 h required	CPR-to-ROSC mean 25.56±15.48 vs 26.00±16.77 min	Target 33°C within 6 h of arrest; rewarming 1°C/day to 36°C	Comparator 35.5–37.5°C, partly active; survival at 1 month; PCPC at 6 months

Within each row, group order follows the comparison shown. Values are median (IQR) unless stated otherwise. Definitions of respiratory and asphyxial arrest differ between reports and were not converted into a uniform percentage. Protocol initiation within 6 h is not an observed time-to-target measurement. NR, not reported; CPR, cardiopulmonary resuscitation; ECMO, extracorporeal membrane oxygenation; IHCA, in-hospital cardiac arrest; OHCA, out-of-hospital cardiac arrest; ROSC, return of spontaneous circulation.

Supplementary Table S4. All 71 excluded reports and reasons

No.	Excluded report	Exclusion category
1	Abend N, Topjian A, Donnelly M, Ichord R, Helfaer M, Nadkarni V, et al. Electrographic seizures are common during therapeutic hypothermia after pediatric cardiac arrest. Ann Neurol 2008;64:S84.	Outside review scope
2	Abend NS, Dlugos D, Herman S, Topjian A, Donnelly M, Ichord R, et al. Continuous EEG monitoring during therapeutic hypothermia after pediatric cardiac arrest. Epilepsia 2008;49:1.	Outside review scope
3	Abend NS, Topjian A, Ichord R, Herman ST, Helfaer M, Donnelly M, et al. Electroencephalographic monitoring during hypothermia after pediatric cardiac arrest. Neurology 2009;72(22):1931–40.	Outside review scope
4	Abend NS, Kessler S, Topjian AA, Gutierrez-Colina AM, Ichord RN, Donnelly M, et al. Short-term outcome prediction by electroencephalographic features in children treated with therapeutic hypothermia after cardiac arrest. Epilepsy Curr 2011;11(1).	Outside review scope
5	Abend NS, Topjian AA, Kessler SK, Gutierrez-Colina AM, Berg RA, Nadkarni V, et al. Outcome prediction by motor and pupillary responses in children treated with therapeutic hypothermia after cardiac arrest. Pediatr Crit Care Med 2012;13(1):32–8.	Outside review scope
6	Bello L, Cruz E, Manganaro S, Andriola M. Analysis of pediatric electroencephalogram characteristics during therapeutic hypothermia in post cardiac arrest patients. Epilepsy Curr 2015;15:63.	Outside review scope
7	Buijs EA, Verboom EM, Top AP, Andrinopoulou ER, Buysse CM, Ince C, et al. Early microcirculatory impairment during therapeutic hypothermia is associated with poor outcome in post-cardiac arrest children: a prospective observational cohort study. Resuscitation 2014;85(3):397–404.	Outside review scope
8	Herrmann J, Fink E, Fabio A, Au A, Berger R, Janesko-Feldman K, et al. Divergent effects of therapeutic hypothermia on cold stress hormones after pediatric cardiac arrest. Crit Care Med 2022;50(1 Suppl):693.	Outside review scope
9	Herrmann JR, Fink EL, Fabio A, Au AK, Berger RP, Janesko-Feldman K, et al. Serum levels of the cold stress hormones FGF21 and GDF-15 after cardiac arrest in infants and children enrolled in single center therapeutic hypothermia clinical trials. Resuscitation 2022;172:173–180.	Outside review scope
10	Kadden M, Zhang A, Shoykhet M. Association of temperature management strategy with fever in critically ill children after out-of-hospital cardiac arrest. Front Pediatr 2024;12:1355385.	Outside review scope
11	Kessler SK, Topjian AA, Gutierrez-Colina AM, Ichord RN, Donnelly M, Nadkarni VM, et al. Short-term outcome prediction by electroencephalographic features in children treated with therapeutic hypothermia after cardiac arrest. Neurocrit Care 2011;14(1):37–43.	Outside review scope
12	Lin JJ, Hsia SH, Wang HS, Chiang MC, Lin KL. Transcranial Doppler ultrasound in therapeutic hypothermia for children after resuscitation. Resuscitation 2015;89:182–7.	Outside review scope
13	Lin JJ, Lin YJ, Hsia SH, Kuo HC, Wang HS, Hsu MH, et al. Early Clinical Predictors of Neurological Outcome in Children With Asphyxial Out-of-Hospital Cardiac Arrest Treated With Therapeutic Hypothermia. Front Pediatr 2020;7:534.	Outside review scope
14	Lin JJ, Hsu MH, Hsia SH, Lin YJ, Wang HS, Kuo HC, et al. Epileptiform discharge and electrographic seizures during the hypothermia phase as predictors of rewarming seizures in children after resuscitation. J Clin Med 2020;9(7):2151.	Outside review scope
15	Manzo AE, Duval-Arnould J, Jones SI, Newton HM, Bembea MM, Hunt EA. Fever in post-cardiac arrest patients treated in the pediatric intensive care unit. Circulation 2017;136.	Outside review scope

No.	Excluded report	Exclusion category
16	McMullin M, Cadotte N, Fuchs E, Kartchner C, Sweney J, Flaherty B. An effective strategy for temperature management in pediatric patients following cardiac arrest. Crit Care Med 2023;51(1):524.	Outside review scope
17	Nenadovic V, Topjian A, Abend N, Dominguez LG, Dlugos D, Berg R, et al. Reduced Variability in Electroencephalographic Spatial Synchrony Patterns During Therapeutic Hypothermia Following Pediatric Cardiac Arrest is Associated with Unfavorable Short-Term Neurological Outcome. Circulation 2009;120(18):S1481.	Outside review scope
18	Prajongkit T, Veeravigrom M, Samransamruajkit R. Prognostic value of continuous electroencephalography in children undergoing therapeutic hypothermia after cardiac arrest: A pilot study. Neurophysiol Clin 2019;49(1):41–47.	Outside review scope
19	van Zellem L, de Jonge R, van Rosmalen J, Reiss I, Tibboel D, Buysse C. High cumulative oxygen levels are associated with improved survival of children treated with mild therapeutic hypothermia after cardiac arrest. Resuscitation 2015;90:150–7.	Outside review scope
20	Yuknis M, Teagarden A, Lutfi R, Rowan C. Targeted temperature management reduces fever incidence in pediatric TBI and cardiac arrest. Crit Care Med 2016;44(12):381.	Outside review scope
21	Zane NR, Reedy MD, Gastonguay MR, Himebauch AS, Ramsey EZ, Topjian AA, et al. A Population Pharmacokinetic Analysis to Study the Effect of Therapeutic Hypothermia on Vancomycin Disposition in Children Resuscitated From Cardiac Arrest. Pediatr Crit Care Med 2017;18(7):e290–e297.	Outside review scope
22	Balcarcel D, Keim G, Mehta SD, Kirschen M, Topjian A, Yehya N. Heterogeneity of Treatment Effect Modeling Identifies Children Who May Benefit From Hypothermia Post-cardiac Arrest. Am J Respir Crit Care Med 2025;211(Suppl 1):A7692.	Ineligible report type
23	Buijs EAB, Verboom EM, Mooij MG, Zwiers AJM, Buysse CMP, Ince C, et al. Early microcirculatory alterations after start of therapeutic hypothermia are associated with mortality in post-cardiac arrest children. Intensive Care Med 2013;39:S21.	Ineligible report type
24	Buttram S, Large B, Yeakel K, Williams K, Bobrow B, Dalton H. Therapeutic hypothermia after pediatric out of hospital cardiac arrest - A single center experience. Crit Care Med 2009;37(12):A256.	Ineligible report type
25	Buttram S, Garcia-Filion P, Williams K, Bobrow B, Dalton H. Hypothermia therapy following pediatric cardiac arrest: Clinical factors correlating with favorable neurologic outcomes. Crit Care Med 2010;38:A132.	Ineligible report type
26	Fink E, Fabio A, Panigrahy A, Berger R, Clark R, Angus D, et al. Exploratory study of duration of hypothermia for neuroprotection after pediatric cardiac arrest. Crit Care Med 2015;43(12):48.	Ineligible report type
27	Kethireddy P, Agarwal N. The effect of therapeutic hypothermia on neurological outcomes in a pediatric submersion injury. Pediatr Crit Care Med 2024;25(11):e88–e89.	Ineligible report type
28	Lin JJ, Hsia SH, Chan OW, Lin KL. 72-hour therapeutic hypothermia improved survival and neurological outcome in pediatric asphyxial out-of-hospital cardiac arrest. Pediatr Crit Care Med 2018;19(6):52.	Ineligible report type
29	Meert K, Telford R, Holubkov R, Slomine B, Christensen J, Dean JM, et al. Safety and efficacy of therapeutic hypothermia after near-miss sudden unexpected infant death. Crit Care Med 2016;44(12):143.	Ineligible report type
30	Moler F, Dean JM. Therapeutic hypothermia after pediatric cardiac arrest in hospital trial. Crit Care Med 2016;44(12):89.	Ineligible report type

No.	Excluded report	Exclusion category
31	Moler FW, Dean JM. Therapeutic hypothermia after pediatric cardiac arrest (THAPCA): out of hospital trial. Pediatric Academic Societies Annual Meeting; San Diego, CA, USA, 25–28 April 2015.	Ineligible report type
32	Namba T, Ohshimo S, Nishikimi M, Miyoshi H, Masuka Y, Shime N. Is Effect Size of Targeted Temperature Management Different for Pediatric Post-Cardiac Arrest Syndrome According to the Severity? Circulation 2023;148(Suppl 1):147.	Ineligible report type
33	Piastra M, Genovese O, Biasucci DG, De Luca D, Pizza A, Trotta R, et al. Therapeutic hypothermia after cardiopulmonary arrest in paediatrics: A prospective series. Intensive Care Med 2013;39:S199.	Ineligible report type
34	Priya B. Association of Hypothermia With Neurological Outcomes In Pediatric Patients After eCPR. ASAIO J 2022;68:75.	Ineligible report type
35	Puiman P, Van Zellem L, De Jager E, Reiss I, Buysse C. Hyperoxia in children treated with therapeutic hypothermia after cardiac arrest: Does it increase mortality? Intensive Care Med 2013;39:S177.	Ineligible report type
36	Ringe H, Reich S, Gratopp A, Varnholt V, Krude H. Goal directed therapeutic hypothermia by dop-pler-ultrasound after CPR in children. A pilot study. Pediatr Crit Care Med 2011;12(3):A24.	Ineligible report type
37	Ringe H, Varnholt V, Reich S, Gratopp A, Reiss A, Kubinski A, et al. Therapeutic hypothermia guidance by cerebral perfusion control by dopplerultrasound after CPR in children. Pediatr Res 2011;70:205.	Ineligible report type
38	Scholefield BR, Morris KP, Duncan HP, Perkins GD, Gosney J, Sanders V, et al. Comparison of the use of therapeutic hypothermia with normothermia after paediatric out-ofhospital cardiac arrest. Pediatr Crit Care Med 2014;15(4):83–84.	Ineligible report type
39	Yunge M, Cordero J, Munoz D, Arrau L, Diaz B, Castro M, et al. Prospective, experimental, multicenter study of hypothermia in post cardiac arrest syndrome in children. Comparison of two methods: Preliminary data. Pediatr Crit Care Med 2014;15(4):6–7.	Ineligible report type
40	Balcarcel DR, Keim GP, Mehta SD, Harhay MO, Himes BE, van Amsterdam WAC, et al. Individualized Treatment Effects of Therapeutic Hypothermia in Children Postcardiac Arrest: A Reanalysis of Two Randomized Clinical Trials. Pediatr Crit Care Med 2026;27(9):1148–1158.	Ineligible report type
41	Harhay MO, Blette BS, Granholm A, Moler FW, Zampieri FG, Goligher EC, et al. A Bayesian Interpretation of a Pediatric Cardiac Arrest Trial (THAPCA-OH). NEJM Evid 2023;2(1):EVIDoa2200196.	Ineligible report type
42	Meert K, Telford R, Holubkov R, Slomine BS, Christensen JR, Dean JM, et al. Exploring the safety and efficacy of targeted temperature management amongst infants with out-of-hospital cardiac arrest due to apparent life threatening events. Resuscitation 2016;109:40–48.	Ineligible report type
43	Moler FW, Hutchison JS, Nadkarni VM, Silverstein FS, Meert KL, Holubkov R, et al. Targeted Temperature Management After Pediatric Cardiac Arrest Due To Drowning: Outcomes and Complications. Pediatr Crit Care Med 2016;17(8):712–20.	Ineligible report type
44	Moler FW, Silverstein FS, Nadkarni VM, Meert KL, Shah SH, Slomine B, et al. Pediatric out-of-hospital cardiac arrest: Time to goal target temperature and outcomes. Resuscitation 2019;135:88–97.	Ineligible report type
45	Rodríguez EA. Therapeutic hypothermia after out-of-hospital cardiac arrest in children. Arch Argent Pediatr 2015;113(6):E368–E369.	Ineligible report type
46	Schmidt CD, Carlton EF, Page K, Hunt NR, Kohne JG, Slomine BS, et al. Association of serum lactate with outcome after pediatric out-of-hospital cardiac arrest: a secondary analysis of the Therapeutic Hypothermia After Pediatric Cardiac Arrest (THAPCA) trial. Resuscitation 2026;226:111178.	Ineligible report type

No.	Excluded report	Exclusion category
47	Scholefield BR, Silverstein FS, Telford R, Holubkov R, Slomine BS, Meert KL, et al. Therapeutic hypothermia after paediatric cardiac arrest: Pooled randomized controlled trials. Resuscitation 2018;133:101–107.	Ineligible report type
48	Slomine BS, Nadkarni VM, Christensen JR, Silverstein FS, Telford R, Topjian A, et al. Pediatric cardiac arrest due to drowning and other respiratory etiologies: Neurobehavioral outcomes in initially comatose children. Resuscitation 2017;115:178–184.	Ineligible report type
49	Slomine BS, Silverstein FS, Page K, Holubkov R, Christensen JR, Dean JM, et al. Relationships between three and twelve month outcomes in children enrolled in the therapeutic hypothermia after pediatric cardiac arrest trials. Resuscitation 2019;139:329–336.	Ineligible report type
50	Topjian AA, Moler FW, Telford R, Holubkov R, Nadkarni VM, Berg RA, et al. Association of early postresuscitation hypotension with survival to discharge after targeted temperature management for pediatric out-of-hospital cardiac arrest secondary analysis of a randomized clinical trial. JAMA Pediatrics 2018;172(2):143–153.	Ineligible report type
51	Topjian AA, Telford R, Holubkov R, Nadkarni VM, Berg RA, Dean JM, et al. The association of early post-resuscitation hypotension with discharge survival following targeted temperature management for pediatric in-hospital cardiac arrest. Resuscitation 2019;141:24–34.	Ineligible report type
52	Abend NS, Topjian A, Ichord R, Herman ST, Helfaer M, Donnelly M, et al. ELECTROENCEPHALOGRAPHIC MONITORING DURING HYPOTHERMIA AFTER PEDIATRIC CARDIAC ARREST Reply. Neurology 2010;74(5):438–439.	No comparative estimate
53	Bustos R. [Therapeutic hypothermia after pediatric cardiac arrest]. An Pediatr (Barc) 2012;76(2):98–102.	No comparative estimate
54	Freire-Gómez X, López-Fernandez E, Aymerich-De-Franceschi C, Gómez-Pastrana-Pau I, Mosquera-Fernandez P, Arias-Dachary J, et al. Effect of deep hypothermia on short-term neurological outcomes in infants with prolonged E-CPR. Cor Vasa 2024;66:8.	No comparative estimate
55	Gupta C, Parkins K. Therapeutic hypothermia and temperature maintenance during post paediatric cardiac arrest transfers: How good are we? Intensive Care Med 2013;39:S173.	No comparative estimate
56	Hoffmann F, Gräsner JT, Brenner S. Implementation of paediatric resuscitation and therapeutic hypothermia registry. Anasthesiol Intensivmed 2011;52(11):727–729.	No comparative estimate
57	Ito A, Mishima Y, Koga Y, Saho M, Hiraki T, Ushijima K. The use of amplitude-integrated electroencephalography combined with continuous conventional electroencephalography during therapeutic hypothermia for an infant with postnatal cardiac arrest. SpringerPlus 2014;3(1):1–4.	No comparative estimate
58	Kim JJ, Kim WJ, Jang JH, Yang HJ, Hyun SY. Implementation of therapeutic hypothermia after pediatric out-of-hospital cardiac arrest in one tertiary emergency center. J Emerg Med 2012;43(5):946.	No comparative estimate
59	Kobr J, Pizingerova K, Sasek L, Fremuth J, Fikrlova S. Induced therapeutic hypothermia following cardiac arrest in children. Bratisl Lek Listy 2011;112(2):92–6.	No comparative estimate
60	Piastra M, Biasucci DG, Picconi E, Pizza A, Genovese O, De Luca D, et al. Therapeutic hypothermia after cardiopulmonary arrest in neonates and children: A prospective series. Resuscitation 2014;85:S99–S100.	No comparative estimate
61	Ringe H, Varnholt V. Therapeutic hypothermia guidance by cerebral perfusion control by dopplerultrasound after CPR in children. Intensive Care Med 2011;37:S384.	No comparative estimate

No.	Excluded report	Exclusion category
62	Topjian AA, Dominguez T, Boyce J, Ichord R, Helfaer M, Nadkarni V. Surface cooling induced therapeutic hypothermia following pediatric cardiac arrest. Circulation 2007;116(16):945.	No comparative estimate
63	Yunge M, Cordero J, Martinez D, Bustos R, Wegner A, Castro M, et al. Effectiveness of Two Targeted Temperature Management Methods After Pediatric Postcardiac Arrest: A Multicenter International Study. Pediatr Crit Care Med 2019;20(2):e77–e82.	No comparative estimate
64	Chang I, Kwak YH, Shin SD, Ro YS, Lee EJ, Ahn KO, et al. Therapeutic hypothermia and outcomes in paediatric out-of-hospital cardiac arrest: A nationwide observational study. Resuscitation 2016;105:8–15.	Unclassifiable exposure
65	Doherty DR, Parshuram CS, Gaboury I, Hoskote A, Lacroix J, Tucci M, et al. Hypothermia therapy after pediatric cardiac arrest. Circulation 2009;119(11):1492–500.	Unclassifiable exposure
66	Magee A, Deschamps R, Delzoppo C, Pan KC, Butt W, Dagan M, et al. Temperature Management and Health-Related Quality of Life in Children 3 Years After Cardiac Arrest. Pediatr Crit Care Med 2022;23(1):13–21.	Unclassifiable exposure
67	Matsui S, Hirayama A, Kitamura T, Sobue T, Hayashi T, Takei H, et al. Target Temperature Management and Survival with Favorable Neurological Outcome After Out-of-Hospital Cardiac Arrest in Children: A Nationwide Multicenter Prospective Study in Japan. Ther Hypothermia Temp Manag 2022;12(1):16–23.	Unclassifiable exposure
68	Sanford EL, Bhaskar P, Li X, Thiagarajan R, Raman L. Hypothermia after extracorporeal cardiopulmonary resuscitation not associated with improved neurologic complications or survival in children: An analysis of the ELSO registry. Resuscitation 2023;188:109852.	Unclassifiable exposure
69	Hickey RW, Kochanek PM, Ferimer H, Graham SH, Safar P. Hypothermia and hyperthermia in children after resuscitation from cardiac arrest. Pediatrics 2000;106(1 I):118–122.	Unclassifiable exposure
70	Morris MC, Helfaer MA, Nadkarni VM. Spontaneous hypothermia and efficacy of interventions to prevent hyperthermia following pediatric cardiopulmonary resuscitation. Crit Care Med 2002;30(12):A119.	Unclassifiable exposure
71	Cheng HH, Rajagopal SK, Sansevere AJ, McDavitt E, Wigmore D, Mecklosky J, et al. Post-arrest therapeutic hypothermia in pediatric patients with congenital heart disease. Resuscitation 2018;126:83–89.	Unclassifiable exposure

Category counts: Outside review scope, n=21; Ineligible report type, n=30; No comparative estimate, n=12; Unclassifiable exposure, n=8.

Categories indicate the principal exclusion reason and match Figure 1. Outside review scope denotes reports without an eligible comparative outcome contrast; duration efficacy need not be the primary question of an otherwise eligible study. Ineligible report type includes abstracts or precursor reports and secondary or overlapping reports. Unclassifiable exposure includes unassignable duration, spontaneous cooling, or mixed nonextractable exposure.

Supplementary Table S5A. Arm-level survival data and individual-study estimates

Study	Analysis role	Time point	Outcome	Intervention, events/total	Comparator, events/total	RR (95% CI)
Scholefield et al., 2015 [8]	Primary	Hospital discharge	Survival to hospital discharge	HT-24 h 13/38	Control 8/35	1.50 (0.71–3.17)
THAPCA-OH, 2015 [4]	Primary	12 months	Alive at 1 year	HT-48 h 57/151	Control 39/136	1.32 (0.94–1.84)
THAPCA-IH, 2017 [5]	Primary	12 months	Alive at 1 year	HT-48 h 81/166	Control 74/161	1.06 (0.84–1.33)
Lin et al., 2018 [11]	Primary	1 month	Alive at 1 month	HT-72 h 15/25	Control 12/39	1.95 (1.10–3.45)
Fink et al., 2018 [7]	Primary	6 months	Alive at 6 months	HT-24 h 9/17	HT-72 h 13/17	0.69 (0.41–1.16)
Fink et al., 2010 [9]	Expanded sensitivity	Hospital discharge	Survival to hospital discharge	HT-24 h* 18/40	Control 63/141	1.01 (0.68–1.49)
Namba et al., 2025 [17]	Expanded sensitivity	30 days	Alive at 30 days	HT-24 h* 53/91	Control 67/216	1.88 (1.44–2.45)
Lin et al., 2013 [10]	Replacement sensitivity	Hospital discharge	Survival to hospital discharge	HT-72 h 11/14	Control 13/28	1.69 (1.04–2.74)

Supplementary Table S5B. Arm-level neurologic outcome data and individual-study estimates

Study	Analysis role	Time point	Outcome	Intervention, events/total	Comparator, events/total	RR (95% CI)
THAPCA-OH, 2015 [4]	Primary	12 months	Alive with VABS-II ≥70	HT-48 h 27/138	Control 15/122	1.59 (0.89–2.85)
THAPCA-IH, 2017 [5]	Primary	12 months	Alive with VABS-II ≥70	HT-48 h 48/133	Control 48/124	0.93 (0.68–1.28)
Lin et al., 2018 [11]	Primary	6 months	Alive with PCPC ≤2	HT-72 h 7/25	Control 1/39	10.92 (1.43–83.50)
Fink et al., 2018 [7]	Primary	6 months	Favorable neurologic outcome	HT-24 h 6/17	HT-72 h 7/17	0.86 (0.36–2.02)
Namba et al., 2025 [17]	Expanded sensitivity	30 days	CPC/PCPC 1–2	HT-24 h* 26/91	Control 23/216	2.68 (1.62–4.44)
Lin et al., 2013 [10]	Replacement sensitivity	Hospital discharge	Alive with PCPC ≤2	HT-72 h 9/14	Control 6/28	3.00 (1.34–6.74)

Study-specific risk ratios were calculated from the displayed favorable-event counts using the conventional log-risk-ratio standard error without a continuity correction. Values greater than 1 favor the intervention node. Control denotes the study-specific comparator strategy and does not represent a uniform protocol. Bracketed reference numbers correspond to the main manuscript reference list.

Fink et al. (2018) counts were derived as complements of reported mortality or unfavorable outcomes. Neurologic counts from Lin et al. were harmonized as all-patient favorable outcomes, with deaths classified as unfavorable. *The HT-24 h classification was approximate in Fink et al. (2010; median 24 h, IQR 16–48 h) and pragmatic in Namba et al. (2025; approximately 24 h, with normothermia in 26 of 91 temperature-managed patients); these studies contributed only to expanded sensitivity analyses. Lin et al. (2013) replaced the potentially overlapping 2018 report in the replacement sensitivity analysis. The Lin 2018 source reported favorable outcomes as 7/15 and 1/12 among 1-month survivors; 7/25 and 1/39 include all enrolled children and classify deaths as unfavorable. Fink 2018 favorable PCPC counts (6/17 and 7/17) are complements of PCPC 4–6 or a >1-point deterioration from baseline. THAPCA neurologic populations exclude baseline-ineligible participants and participants without evaluable outcomes; they are not all randomized denominators.

CI, confidence interval; CPC, Cerebral Performance Category; h, hours; HT, hypothermia; PCPC, Pediatric Cerebral Performance Category; RR, risk ratio; THAPCA-IH, Therapeutic Hypothermia After Pediatric Cardiac Arrest—in-hospital trial; THAPCA-OH, Therapeutic Hypothermia After Pediatric Cardiac Arrest—out-of-hospital trial; VABS-II, Vineland Adaptive Behavior Scales, Second Edition.

Supplementary Table S6. CINeMA certainty assessment — A. Survival

Comparison	RR (95% CI)	Direct studies, n	Within-study bias	Reporting bias	Indirectness	Imprecision	Heterogeneity	Incoherence	Confidence
HT-24 h vs Control	1.42 (0.83–2.44)	1	High risk	Some concerns	Some concerns	Major concerns	No concerns	No concerns	Very low
HT-48 h vs Control	1.14 (0.94–1.37)	2	Low risk	Low risk	No concerns	Some concerns	Some concerns	No concerns	Moderate
HT-72 h vs Control	2.01 (1.24–3.25)	1	High risk	Some concerns	Major concerns	No concerns	Major concerns	No concerns	Very low
HT-48 h vs HT-24 h	0.80 (0.45–1.41)	0	Some concerns	Some concerns	Some concerns	Major concerns	No concerns	No concerns	Very low
HT-72 h vs HT-24 h	1.41 (0.89–2.22)	1	Some concerns	Some concerns	No concerns	Some concerns	Some concerns	No concerns	Low
HT-72 h vs HT-48 h	1.77 (1.05–2.97)	0	Some concerns	Some concerns	Some concerns	No concerns	Major concerns	No concerns	Very low

Confidence reflects joint interpretation of the six domains, with correlated concerns considered together rather than mechanically summed. The clinical-importance range of RR 0.833–1.20 was selected post hoc. Under CINeMA rules, a confidence interval entirely on one side of RR=1 can receive no concerns for imprecision; this does not imply precise estimation of the effect magnitude. CINeMA, Confidence in Network Meta-Analysis; CI, confidence interval; HT, hypothermia; RR, risk ratio. The CINeMA method is described in reference [25] of the main manuscript.

Supplementary Table S6. CINeMA certainty assessment — B. Favorable neurologic outcome

Comparison	RR (95% CI)	Direct studies, n	Within-study bias	Reporting bias	Indirectness	Imprecision	Heterogeneity	Incoherence	Confidence
HT-24 h vs Control	9.36 (0.89–98.38)	0	Major concerns	Some concerns	Some concerns	Some concerns	Some concerns	Major concerns	Very low
HT-48 h vs Control	1.15 (0.69–1.92)	2	Some concerns	Low risk	No concerns	Major concerns	Some concerns	Major concerns	Very low
HT-72 h vs Control	10.92 (1.32–90.40)	1	Major concerns	Some concerns	Major concerns	No concerns	Some concerns	Major concerns	Very low
HT-48 h vs HT-24 h	0.12 (0.01–1.36)	0	Major concerns	Some concerns	Some concerns	Major concerns	Some concerns	Major concerns	Very low
HT-72 h vs HT-24 h	1.17 (0.42–3.28)	1	Major concerns	Some concerns	No concerns	Major concerns	Some concerns	Major concerns	Very low
HT-72 h vs HT-48 h	9.50 (1.08–83.61)	0	Major concerns	Some concerns	Some concerns	No concerns	Some concerns	Major concerns	Very low

Confidence reflects joint interpretation of the six domains, with correlated concerns considered together rather than mechanically summed. The clinical-importance range of RR 0.833–1.20 was selected post hoc. Under CINeMA rules, a confidence interval entirely on one side of RR=1 can receive no concerns for imprecision; this does not imply precise estimation of the effect magnitude. In the neurologic network, incoherence could not be tested because there were no closed loops. Major concerns were assigned according to CINeMA rules for this situation, not because incoherence had been demonstrated. CINeMA, Confidence in Network Meta-Analysis; CI, confidence interval; HT, hypothermia; RR, risk ratio. The CINeMA method is described in reference [25] of the main manuscript.

Supplementary Table S7A. Influence, restriction, and denominator analyses

Analysis	Comparison	Estimate or finding	Interpretation
Omit Lin 2018—survival	HT-72 h vs Control	RR 2.16 (95% CI 0.86–5.43)	Statistical significance removed
Omit Lin 2018—survival	HT-72 h vs HT-48 h	RR 1.90 (95% CI 0.74–4.87)	Statistical significance removed
Omit Lin 2018—neurologic	HT-72 h vs Control and HT-48 h	Not estimable	Network disconnected; HT-72 h vs HT-24 h remains estimable
Survival ≥6 months	HT-48 h vs Control	RR 1.14 (95% CI 0.93–1.39)	THAPCA forms one component; Fink forms a separate duration component
Survival ≥6 months	HT-72 h vs HT-24 h	RR 1.44 (95% CI 0.86–2.43)	Single small pilot RCT
Full randomized denominator—neurologic	HT-72 h vs Control	RR 10.92 (95% CI 1.34–88.89)	Direction preserved; extreme imprecision
Full randomized denominator—neurologic	HT-72 h vs HT-48 h	RR 9.18 (95% CI 1.07–78.87)	Indirect; extreme imprecision
Prediction intervals—survival	All comparisons	All crossed RR=1	Future-study effects remain uncertain
Prediction intervals—neurologic	All comparisons	Not estimable	Network too sparse
Heterogeneity	Survival	I^2=0%	No detected statistical heterogeneity; clinical heterogeneity remains
Heterogeneity	Neurologic	I^2=60%	Uncertain estimate informed only by the two THAPCA trials
Node splitting	HT-72 h vs HT-48 h	Indirect only	No direct–indirect comparison possible
RCT-only/strict comparator networks	Four-node network	Disconnected	HT-72 h vs Control or HT-48 h not estimable; HT-72 h vs HT-24 h remains estimable

Omission of Lin 2018 is distinct from replacement by Lin 2013. Disconnection means a contrast is not estimable; it does not demonstrate absence of a treatment effect. RCT-only and strict protocolized active-normothermia restrictions retain THAPCA-OH, THAPCA-IH, and Fink 2018, yielding separate HT-48 h–Control and HT-24 h–HT-72 h components. Expanded and replacement analyses are those displayed in Figure 4.

Supplementary Table S7B. Common-effect versus random-effects RR models

Outcome	Comparison	Random-effects RR (95% CI)	Common-effect RR (95% CI)
Survival	HT-24 h vs Control	1.42 (0.83–2.44)	1.42 (0.83–2.44)
Survival	HT-48 h vs Control	1.14 (0.94–1.37)	1.14 (0.94–1.37)
Survival	HT-72 h vs Control	2.01 (1.24–3.25)	2.01 (1.24–3.25)
Survival	HT-48 h vs HT-24 h	0.80 (0.45–1.41)	0.80 (0.45–1.41)
Survival	HT-72 h vs HT-24 h	1.41 (0.89–2.22)	1.41 (0.89–2.22)
Survival	HT-72 h vs HT-48 h	1.77 (1.05–2.97)	1.77 (1.05–2.97)
Favorable neurologic outcome	HT-24 h vs Control	9.36 (0.89–98.38)	9.36 (1.03–85.15)
Favorable neurologic outcome	HT-48 h vs Control	1.15 (0.69–1.92)	1.05 (0.80–1.39)
Favorable neurologic outcome	HT-72 h vs Control	10.92 (1.32–90.40)	10.92 (1.43–83.50)
Favorable neurologic outcome	HT-48 h vs HT-24 h	0.12 (0.01–1.36)	0.11 (0.01–1.04)
Favorable neurologic outcome	HT-72 h vs HT-24 h	1.17 (0.42–3.28)	1.17 (0.49–2.75)
Favorable neurologic outcome	HT-72 h vs HT-48 h	9.50 (1.08–83.61)	10.37 (1.33–80.79)

Common-effect models assume no between-study heterogeneity. Survival results coincide after rounding; neurologic intervals depend on specification. CI, confidence interval; HT, hypothermia; RR, risk ratio.

Supplementary Table S7C. Mantel–Haenszel common-effect OR models

Outcome	Comparison	Inverse-variance common RR (95% CI)	Mantel–Haenszel common OR (95% CI)
Survival	HT-24 h vs Control	1.42 (0.83–2.44)	1.59 (0.65–3.90)
Survival	HT-48 h vs Control	1.14 (0.94–1.37)	1.28 (0.92–1.77)
Survival	HT-72 h vs Control	2.01 (1.24–3.25)	3.74 (1.51–9.27)
Survival	HT-48 h vs HT-24 h	0.80 (0.45–1.41)	0.80 (0.31–2.09)
Survival	HT-72 h vs HT-24 h	1.41 (0.89–2.22)	2.36 (0.83–6.68)
Survival	HT-72 h vs HT-48 h	1.77 (1.05–2.97)	2.94 (1.12–7.69)
Favorable neurologic outcome	HT-24 h vs Control	9.36 (1.03–85.15)	11.52 (0.88–151.11)
Favorable neurologic outcome	HT-48 h vs Control	1.05 (0.80–1.39)	1.14 (0.76–1.70)
Favorable neurologic outcome	HT-72 h vs Control	10.92 (1.43–83.50)	14.78 (1.69–129.30)
Favorable neurologic outcome	HT-48 h vs HT-24 h	0.11 (0.01–1.04)	0.10 (0.01–1.34)
Favorable neurologic outcome	HT-72 h vs HT-24 h	1.17 (0.49–2.75)	1.28 (0.32–5.13)
Favorable neurologic outcome	HT-72 h vs HT-48 h	10.37 (1.33–80.79)	13.01 (1.43–118.14)

The netmeta Mantel–Haenszel implementation estimates ORs. ORs and RRs are different effect measures and must not be compared for numerical magnitude. This check concerns direction and interval inclusion of the null. Sparse events and common-effect assumptions remain limitations. OR, odds ratio.

Supplementary Table S7D. Random-effects prediction intervals

Outcome	Comparison	RR (95% CI)	95% prediction interval
Survival	HT-24 h vs Control	1.42 (0.83–2.44)	0.44–4.64
Survival	HT-48 h vs Control	1.14 (0.94–1.37)	0.75–1.72
Survival	HT-72 h vs Control	2.01 (1.24–3.25)	0.69–5.80
Survival	HT-48 h vs HT-24 h	0.80 (0.45–1.41)	0.23–2.79
Survival	HT-72 h vs HT-24 h	1.41 (0.89–2.22)	0.52–3.83
Survival	HT-72 h vs HT-48 h	1.77 (1.05–2.97)	0.57–5.51
Favorable neurologic outcome	HT-24 h vs Control	9.36 (0.89–98.38)	Not estimable
Favorable neurologic outcome	HT-48 h vs Control	1.15 (0.69–1.92)	Not estimable
Favorable neurologic outcome	HT-72 h vs Control	10.92 (1.32–90.40)	Not estimable
Favorable neurologic outcome	HT-48 h vs HT-24 h	0.12 (0.01–1.36)	Not estimable
Favorable neurologic outcome	HT-72 h vs HT-24 h	1.17 (0.42–3.28)	Not estimable
Favorable neurologic outcome	HT-72 h vs HT-48 h	9.50 (1.08–83.61)	Not estimable

All survival intervals include RR=1. Neurologic intervals cannot be estimated with the available residual degrees of freedom. The wide survival intervals also reflect the very small network; they should not be interpreted as validating transitivity.

Supplementary Table S7E. Direct, indirect, and network estimates in the survival loop

Comparison	Direct RR (95% CI)	Indirect RR (95% CI)	Network RR (95% CI)	P for disagreement
HT-24 h vs Control	1.50 (0.71–3.17)	1.35 (0.62–2.92)	1.42 (0.83–2.44)	0.851
HT-72 h vs Control	1.95 (1.10–3.45)	2.16 (0.87–5.39)	2.01 (1.24–3.25)	0.851
HT-24 h vs HT-72 h	0.69 (0.41–1.16)	0.77 (0.30–1.97)	0.71 (0.45–1.12)	0.851

The HT-24 h versus HT-72 h row is oriented oppositely to HT-72 h versus HT-24 h in the main results. The three tests reflect one survival loop and are not independent confirmations of consistency. HT-72 h versus HT-48 h is entirely indirect and cannot undergo a direct–indirect disagreement test. No loop is present for neurologic outcome.

Supplementary Table S7F. THAPCA neurologic populations and denominator sensitivity

Trial / arm	Randomized	Baseline eligible	Baseline ineligible	Evaluable neurologic	Favorable events	Eligible unknown vital status	Eligible alive, missing VABS
THAPCA-OH 2015 / HT-48 h	155	142	13	138	27	4	0
THAPCA-OH 2015 / Control	140	128	12	122	15	4	2
THAPCA-IH 2017 / HT-48 h	166	135	31	133	48	0	2
THAPCA-IH 2017 / Control	163	134	29	124	48	2	8

Comparison	Primary RR (95% CI)	Full randomized denominator RR (95% CI)
HT-24 h vs Control	9.36 (0.89–98.38)	9.36 (0.92–95.45)
HT-48 h vs Control	1.15 (0.69–1.92)	1.19 (0.74–1.92)
HT-72 h vs Control	10.92 (1.32–90.40)	10.92 (1.34–88.89)
HT-48 h vs HT-24 h	0.12 (0.01–1.36)	0.13 (0.01–1.36)
HT-72 h vs HT-24 h	1.17 (0.42–3.28)	1.17 (0.43–3.16)
HT-72 h vs HT-48 h	9.50 (1.08–83.61)	9.18 (1.07–78.87)

The sensitivity analysis retains observed favorable-event numerators and uses 155/140 (THAPCA-OH) and 166/163 (THAPCA-IH) as denominators. It assumes unfavorable outcomes for every participant without an observed favorable outcome, including baseline-ineligible participants as well as losses to follow-up. Baseline ineligibility is not missing follow-up. This is an assumption-based estimand sensitivity analysis, not a fully observed intention-to-treat analysis.

Supplementary Table S8. Reported adverse events and safety outcomes

Study	Arm A	Arm B	Safety outcome	Arm A result	Arm B result	Statistical result or note
Moler 2015 THAPCA-OH	HT-48 h (n=153)	Active normothermia (n=139)	Serious arrhythmia	17/153 (11%)	13/137 (9%)	P=0.66
Moler 2015 THAPCA-OH	HT-48 h (n=153)	Active normothermia (n=139)	Culture-proven infection	70/153 (46%)	54/137 (39%)	P=0.28
Moler 2015 THAPCA-OH	HT-48 h (n=153)	Active normothermia (n=139)	Any blood product	83/153 (54%)	74/138 (54%)	P=0.92
Moler 2017 THAPCA-IH	HT-48 h (n=161)	Active normothermia (n=160)	Serious arrhythmia	25/161 (16%)	23/160 (14%)	P=0.78
Moler 2017 THAPCA-IH	HT-48 h (n=161)	Active normothermia (n=160)	Culture-proven infection	44/161 (27%)	46/160 (29%)	P=0.78
Moler 2017 THAPCA-IH	HT-48 h (n=161)	Active normothermia (n=160)	Any blood product	139/161 (86%)	140/160 (88%)	P=0.76
Fink 2018	HT-24 h (n=17)	HT-72 h (n=17)	Infection	12%	24%	No significant between-group difference reported
Fink 2018	HT-24 h (n=17)	HT-72 h (n=17)	Seizure	35%	35%	No significant between-group difference reported
Fink 2018	HT-24 h (n=17)	HT-72 h (n=17)	Bleeding	6%	6%	No significant between-group difference reported
Fink 2018	HT-24 h (n=17)	HT-72 h (n=17)	Rearrest	18%	0%	No significant between-group difference reported
Scholefield 2015	HT-24 h (n=38)	Standard temperature management <38 C (n=35)	Bradycardia within 72 h	42%	19%	P=0.04
Scholefield 2015	HT-24 h (n=38)	Standard temperature management <38 C (n=35)	Hypotension within 72 h	63%	28%	P=0.004
Scholefield 2015	HT-24 h (n=38)	Standard temperature management <38 C (n=35)	Inotropic support within 72 h	74%	54%	P=0.08
Scholefield 2015	HT-24 h (n=38)	Standard temperature management <38 C (n=35)	Seizure within 72 h	8%	14%	No significant between-group difference reported

Study	Arm A	Arm B	Safety outcome	Arm A result	Arm B result	Statistical result or note
Fink 2010	HT-24 h* (n=40)	Standard care (n=141)	Intermittent arrhythmia	3/40 (7.5%)	6/141 (4.3%)	P=0.40
Fink 2010	HT-24 h* (n=40)	Standard care (n=141)	Positive culture	24/40 (60.0%)	73/141 (51.8%)	P=0.17
Fink 2010	HT-24 h* (n=40)	Standard care (n=141)	Hemorrhage	1/40 (2.5%)	7/141 (5.0%)	P=1.00
Fink 2010	HT-24 h* (n=40)	Standard care (n=141)	>=4 potassium doses	24/40 (60.0%)	32/141 (22.7%)	Descriptive arm-level result
Fink 2010	HT-24 h* (n=40)	Standard care (n=141)	>=4 magnesium doses	4/40 (10.0%)	1/141 (0.7%)	Descriptive arm-level result
Fink 2010	HT-24 h* (n=40)	Standard care (n=141)	1-3 phosphorus doses	17/40 (42.5%)	22/141 (15.6%)	Descriptive arm-level result
Fink 2010	HT-24 h* (n=40)	Standard care (n=141)	Rearrest	5/40 (12.5%)	36/141 (25.5%)	P=0.09
Lin 2013	Earlier report	Overlapping cohort	Safety outcomes	Not separately extracted	Not separately extracted	Excluded from this descriptive synthesis to avoid double counting with Lin 2018
Lin 2018	HT-72 h (n=25)	Normothermia (n=39)	Bradycardia	13/25 (52.0%)	10/39 (25.6%)	P=0.031
Lin 2018	HT-72 h (n=25)	Normothermia (n=39)	Coagulopathy	11/25 (44.0%)	8/39 (20.5%)	P=0.044
Lin 2018	HT-72 h (n=25)	Normothermia (n=39)	Infection	0/25 (0%)	3/39 (7.7%)	P=0.274
Lin 2018	HT-72 h (n=25)	Normothermia (n=39)	Electrolyte imbalance	13/25 (52.0%)	21/39 (53.8%)	P=0.887
Namba 2025	Mixed 24-h temperature management (n=91)	No temperature management (n=216)	Safety outcomes	NR	NR	No arm-level adverse-event data suitable for extraction; sensitivity analysis only

Note: Values are n/N (%) unless otherwise stated. Outcomes, definitions, and ascertainment windows were not uniform; the table is descriptive and should not be used for indirect cross-study comparisons. NR indicates not reported. HT, hypothermia; IH, in-hospital cardiac arrest; OH, out-of-hospital cardiac arrest; RCT, randomized controlled trial. Safety denominators can differ from randomized or efficacy denominators. Lin 2013 was not extracted separately to avoid double counting with its later report. Events are not consistently reported by duration, and none of these descriptive results establishes a duration-specific causal safety effect. *Fink 2010 had an approximate 24-hour exposure classification, not a fixed intended-duration protocol.

Supplementary Table S9. Ongoing randomized duration trial

Trial and registry	Status	Population	Interventions	Primary outcome	Planned enrollment and timeline	Relevance
P-ICECAP ClinicalTrials.gov NCT05376267	Recruiting (registry accessed 1 October 2026)	Comatose children aged 2 days to <18 years after out-of-hospital cardiac arrest	Initial 1:1:1 randomization to 24-, 48-, and 72-h cooling; subsequent response-adaptive allocation among controlled normothermia (0 h) and cooling for 12-96 h	One-year composite of mortality and Vineland Adaptive Behavior Scales, Third Edition outcome	Up to 900 participants; started August 2022; estimated completion March 2028	Directly addresses the duration question and may provide more definitive evidence than the current sparse network

Note: P-ICECAP, Pediatric Influence of Cooling Duration on Efficacy in Cardiac Arrest Patients; VABS-3, Vineland Adaptive Behavior Scales, Third Edition. Status reflects registry information accessed on October 1, 2026; this status check was separate from the original screening set, and ongoing trials were not included as outcome evidence.

Supplementary Table S10A. Outcome-specific RoB 2 judgments

Trial	Outcome	D1	D2	D3	D4	D5	Overall
THAPCA-OH 2015	Survival	Low	Low	Low	Low	Low	Low
THAPCA-OH 2015	Favorable neurologic outcome	Low	Low	Low	Some concerns	Low	Some concerns
THAPCA-IH 2017	Survival	Low	Low	Low	Low	Low	Low
THAPCA-IH 2017	Favorable neurologic outcome	Low	Low	Low	Some concerns	Low	Some concerns
Fink 2018	Survival	Some concerns	Some concerns	Low	Low	Some concerns	Some concerns
Fink 2018	Favorable neurologic outcome	Some concerns	Some concerns	Low	High	Some concerns	High

RoB 2, August 22, 2019 version. D1, randomization; D2, deviations from intended interventions; D3, missing outcome data; D4, outcome measurement; D5, selection of the reported result. The THAPCA central interviewer was blinded, but caregiver input was not; this supports some concerns for neurologic measurement rather than asserting that central assessors were unblinded. Fink neurologic assessment relied on unblinded clinical assessment, supporting high risk. Survival is an objective outcome. The revised source-based judgments were reviewed and approved by the corresponding author.

Supplementary Table S10B. Outcome-specific ROBINS-I judgments

Study	Outcome	D1	D2	D3	D4	D5	D6	D7	Overall
Fink 2010	Survival	Serious	No information	Moderate	Low	Low	Low	Moderate	Serious
Lin 2013	Survival	Serious	Serious	Moderate	Low	Low	Low	Moderate	Serious
Lin 2013	Favorable neurologic outcome	Serious	Serious	Moderate	Low	Low	Serious	Moderate	Serious
Scholefield 2015	Survival	Serious	Low	Low	Low	Low	Low	Moderate	Serious
Lin 2018	Survival	Serious	Serious	Moderate	Low	Low	Low	Moderate	Serious
Lin 2018	Favorable neurologic outcome	Serious	Serious	Moderate	Low	Low	Serious	Moderate	Serious
Namba 2025	Survival	Serious	No information	Moderate	Low	No information	Low	Moderate	Serious
Namba 2025	Favorable neurologic outcome	Serious	No information	Moderate	Low	No information	Serious	Moderate	Serious

ROBINS-I, original 2016 version. D1, confounding; D2, participant selection; D3, intervention classification; D4, deviations from intended interventions; D5, missing data; D6, outcome measurement; D7, selection of the reported result. Serious confounding risk persists in every observational report. Lin participant selection requires survival ≥12 h and creates potential survivor-selection or immortal-time bias. D3 is moderate for Fink 2010 and Namba 2025 because durations or temperature exposure were mixed. No information indicates insufficient reporting, not automatically serious risk.

Supplementary Table S10C. Principal domain-level rationales

Studies	Domain / outcome	Rationale
THAPCA-OH and THAPCA-IH	D4; survival versus neurologic outcome	Vital status is objective. VABS-II was centrally assessed by blinded interviewers, but unblinded caregivers supplied information that could influence the score. Neurologic D4 was therefore judged some concerns, while survival D4 was low.
Fink 2018	D1, D2, D5; both outcomes	The small pilot trial provides limited detail on allocation and deviations, and selective-result concerns could not be fully excluded. These domains were retained as some concerns.
Fink 2018	D4; neurologic outcome	PCPC assessment involved unblinded clinical evaluation. The influence of knowledge of treatment on the neurologic judgment could not be excluded; high risk was retained.
All observational reports	D1; all assessed outcomes	Nonrandom temperature-strategy selection permits confounding by arrest etiology, severity, resuscitation course, treatment selection, and cointerventions; aggregate data do not remove this risk.
Lin 2013 and Lin 2018	D2; both outcomes	Enrollment required survival for ≥12 h after ROSC. Treatment selection and classification in the observational setting could introduce survivor-selection or immortal-time bias. This does not establish that only children completing all 72 h were analyzed.
Fink 2010 and Namba 2025	D3; assessed outcomes	Fink duration was decided individually and varied around a 24-hour median. Namba mixed hypothermic and normothermic targets, with approximate rather than fixed duration. Intervention classification was therefore moderate risk.
Lin 2013, Lin 2018, and Namba 2025	D6; neurologic outcome	Neurologic categorization in nonrandomized cohorts was vulnerable to unblinded or insufficiently documented outcome assessment. Serious risk was retained for neurologic measurement, while objective survival measurement was low.
Fink 2010 and Namba 2025	Domains with no information	Reporting did not support definitive participant-selection judgments; Namba also lacked enough information for a definitive missing-data judgment. No information is retained rather than substituting an unsupported low or serious judgment.

Supplementary S1. Search strategies

Searches were completed on August 20, 2026. The revised strategy version was dated August 10, 2026; final source-specific executions occurred on August 17–20, 2026. These August executions supplied the reported screening counts. Reference lists of the prior duration network meta-analysis and the recent pairwise review were checked; no additional eligible reports were identified.

S1A. Search sources and reporting fields

Source	Platform	Main search	Supplemental searches	Final execution date	Results / exported
PubMed/MEDLINE	PubMed	Combined controlled-vocabulary + tiab search	Duration; rewarming/maintenance	2026-08-17	1,043 / 1,043
Embase	Embase.com	Emtree + ti,ab,kw search	Duration; rewarming/maintenance	2026-08-17	3,857 / 3,857
Scopus	Scopus document search	TITLE-ABS-KEY combined search	Duration; rewarming/maintenance	2026-08-17	3,250 / 3,250
Web of Science Core Collection	Web of Science	TS= combined search	Duration; rewarming/maintenance	2026-08-20	1,039 / 1,039
Cochrane CENTRAL	Cochrane Library Search Manager	MeSH + ti,ab,kw combined search	Duration; rewarming/maintenance	2026-08-17	168 / 168
ClinicalTrials.gov	Expert Search	Broad combined registry query	None required	2026-08-17	72 / 72

S1B. PubMed/MEDLINE

Platform: PubMed | Final strategy version: 2026-08-10 | Final execution date: 2026-08-17 | Results/exported: 1,043/1,043 records

#1 Cardiac arrest / post-arrest concept

Search query
("Heart Arrest"[Mesh] OR "Out-of-Hospital Cardiac Arrest"[Mesh] OR "Cardiopulmonary Resuscitation"[Mesh] OR "cardiac arrest"[tiab] OR "heart arrest"[tiab] OR "cardiopulmonary arrest"[tiab] OR "cardiorespiratory arrest"[tiab] OR "cardiopulmonary resuscitation"[tiab] OR CPR[tiab] OR ROSC[tiab] OR "return of spontaneous circulation"[tiab] OR "return of circulation"[tiab] OR OHCA[tiab] OR IHCA[tiab] OR "post-arrest"[tiab] OR postarrest[tiab] OR "post cardiac arrest"[tiab] OR "post-cardiac arrest"[tiab] OR postresuscitation[tiab] OR "post-resuscitation"[tiab])

#2 Pediatric concept

Search query
("Infant"[Mesh] OR "Child"[Mesh] OR "Adolescent"[Mesh] OR "Pediatrics"[Mesh] OR pediatric*[tiab] OR paediatric*[tiab] OR child*[tiab] OR infant*[tiab] OR adolescent*[tiab])

#3 Temperature-management concept

Search query
("Hypothermia, Induced"[Mesh] OR "Body Temperature Regulation"[Mesh] OR "targeted temperature management"[tiab] OR TTM[tiab] OR "therapeutic hypothermia"[tiab] OR "induced hypothermia"[tiab] OR "mild hypothermia"[tiab] OR hypothermia[tiab] OR cooling[tiab] OR normothermia[tiab] OR "therapeutic normothermia"[tiab:~0] OR "active normothermia"[tiab:~0] OR "temperature management"[tiab] OR "temperature control"[tiab] OR "controlled temperature"[tiab] OR "temperature target"[tiab] OR "fever prevention"[tiab:~0] OR "fever avoidance"[tiab:~0] OR fever[tiab])

#4 Main combined search

Search query
("Heart Arrest"[Mesh] OR "Out-of-Hospital Cardiac Arrest"[Mesh] OR "Cardiopulmonary Resuscitation"[Mesh] OR "cardiac arrest"[tiab] OR "heart arrest"[tiab] OR "cardiopulmonary arrest"[tiab] OR "cardiorespiratory arrest"[tiab] OR "cardiopulmonary resuscitation"[tiab] OR CPR[tiab] OR ROSC[tiab] OR "return of spontaneous circulation"[tiab] OR "return of circulation"[tiab] OR OHCA[tiab] OR IHCA[tiab] OR "post-arrest"[tiab] OR postarrest[tiab] OR "post cardiac arrest"[tiab] OR "post-cardiac arrest"[tiab] OR postresuscitation[tiab] OR "post-resuscitation"[tiab])<br>AND<br>("Infant"[Mesh] OR "Child"[Mesh] OR "Adolescent"[Mesh] OR "Pediatrics"[Mesh] OR pediatric*[tiab] OR paediatric*[tiab] OR child*[tiab] OR infant*[tiab] OR adolescent*[tiab])<br>AND<br>("Hypothermia, Induced"[Mesh] OR "Body Temperature Regulation"[Mesh] OR "targeted temperature management"[tiab] OR TTM[tiab] OR "therapeutic hypothermia"[tiab] OR "induced hypothermia"[tiab] OR "mild hypothermia"[tiab] OR hypothermia[tiab] OR cooling[tiab] OR normothermia[tiab] OR "therapeutic normothermia"[tiab:~0] OR "active normothermia"[tiab:~0] OR "temperature management"[tiab] OR "temperature control"[tiab] OR "controlled temperature"[tiab] OR "temperature target"[tiab] OR "fever prevention"[tiab:~0] OR "fever avoidance"[tiab:~0] OR fever[tiab])<br>NOT (animals[mh] NOT humans[mh])

This main combined search determines the screening set. Duration is not required.

Final execution note (2026-08-17): the main query returned and exported 1,043 records in PubMed. The temperature-management block was combined with both the pediatric and cardiac-arrest blocks by AND; therefore, fever[tiab] did not retrieve general pediatric fever literature by itself. Narrow terms such as active normothermia and fever prevention/avoidance were retained explicitly even when subsumed by broader terms.

#5 Supplemental duration validation search : 236 / 2026-08-17

Search query
("Heart Arrest"[Mesh] OR "Out-of-Hospital Cardiac Arrest"[Mesh] OR "Cardiopulmonary Resuscitation"[Mesh] OR "cardiac arrest"[tiab] OR "heart arrest"[tiab] OR "cardiopulmonary arrest"[tiab] OR "cardiorespiratory arrest"[tiab] OR "cardiopulmonary resuscitation"[tiab] OR CPR[tiab] OR ROSC[tiab] OR "return of spontaneous circulation"[tiab] OR "return of circulation"[tiab] OR OHCA[tiab] OR IHCA[tiab] OR "post-arrest"[tiab] OR postarrest[tiab] OR "post cardiac arrest"[tiab] OR "post-cardiac arrest"[tiab] OR postresuscitation[tiab] OR "post-resuscitation"[tiab])<br>AND<br>("Infant"[Mesh] OR "Child"[Mesh] OR "Adolescent"[Mesh] OR "Pediatrics"[Mesh] OR pediatric*[tiab] OR paediatric*[tiab] OR child*[tiab] OR infant*[tiab] OR adolescent*[tiab])<br>AND<br>("Hypothermia, Induced"[Mesh] OR "Body Temperature Regulation"[Mesh] OR "targeted temperature management"[tiab] OR TTM[tiab] OR "therapeutic hypothermia"[tiab] OR "induced hypothermia"[tiab] OR "mild hypothermia"[tiab] OR hypothermia[tiab] OR cooling[tiab] OR normothermia[tiab] OR "therapeutic normothermia"[tiab:~0] OR "active normothermia"[tiab:~0] OR "temperature management"[tiab] OR "temperature control"[tiab] OR "controlled temperature"[tiab] OR "temperature target"[tiab] OR "fever prevention"[tiab:~0] OR "fever avoidance"[tiab:~0] OR fever[tiab])<br>AND<br>(duration[tiab] OR "cooling duration"[tiab] OR "treatment duration"[tiab] OR maintenance[tiab] OR "24 hour"[tiab] OR "24 hours"[tiab] OR "24 h"[tiab] OR 24h[tiab] OR "48 hour"[tiab] OR "48 hours"[tiab] OR "48 h"[tiab] OR 48h[tiab] OR "72 hour"[tiab] OR "72 hours"[tiab] OR "72 h"[tiab] OR 72h[tiab])<br>NOT (animals[mh] NOT humans[mh])

#6 Supplemental rewarming/maintenance search : 120 / 2026-08-17

Search query
("Heart Arrest"[Mesh] OR "Out-of-Hospital Cardiac Arrest"[Mesh] OR "Cardiopulmonary Resuscitation"[Mesh] OR "cardiac arrest"[tiab] OR "heart arrest"[tiab] OR "cardiopulmonary arrest"[tiab] OR "cardiorespiratory arrest"[tiab] OR "cardiopulmonary resuscitation"[tiab] OR CPR[tiab] OR ROSC[tiab] OR "return of spontaneous circulation"[tiab] OR "return of circulation"[tiab] OR OHCA[tiab] OR IHCA[tiab] OR "post-arrest"[tiab] OR postarrest[tiab] OR "post cardiac arrest"[tiab] OR "post-cardiac arrest"[tiab] OR postresuscitation[tiab] OR "post-resuscitation"[tiab])<br>AND<br>("Infant"[Mesh] OR "Child"[Mesh] OR "Adolescent"[Mesh] OR "Pediatrics"[Mesh] OR pediatric*[tiab] OR paediatric*[tiab] OR child*[tiab] OR infant*[tiab] OR adolescent*[tiab])<br>AND<br>("Hypothermia, Induced"[Mesh] OR "Body Temperature Regulation"[Mesh] OR "targeted temperature management"[tiab] OR TTM[tiab] OR "therapeutic hypothermia"[tiab] OR "induced hypothermia"[tiab] OR "mild hypothermia"[tiab] OR hypothermia[tiab] OR cooling[tiab] OR normothermia[tiab] OR "therapeutic normothermia"[tiab:~0] OR "active normothermia"[tiab:~0] OR "temperature management"[tiab] OR "temperature control"[tiab] OR "controlled temperature"[tiab] OR "temperature target"[tiab] OR "fever prevention"[tiab:~0] OR "fever avoidance"[tiab:~0] OR fever[tiab])<br>AND<br>(rewarming[tiab] OR re-warming[tiab] OR "rewarming rate"[tiab] OR maintenance[tiab] OR "maintenance phase"[tiab])<br>NOT (animals[mh] NOT humans[mh])

S1C. Embase

Platform: Embase.com | Final strategy version: 2026-08-10 | Final execution date: 2026-08-17 | Results/exported: 3,857/3,857 records

#1 Cardiac arrest / post-arrest concept

**Search query**
('heart arrest'/exp OR 'cardiopulmonary resuscitation'/exp OR 'return of spontaneous circulation'/exp OR 'cardiac arrest':ti,ab,kw OR 'heart arrest':ti,ab,kw OR 'cardiopulmonary arrest':ti,ab,kw OR 'cardiorespiratory arrest':ti,ab,kw OR 'cardiopulmonary resuscitation':ti,ab,kw OR cpr:ti,ab,kw OR rosc:ti,ab,kw OR 'return of spontaneous circulation':ti,ab,kw OR 'return of circulation':ti,ab,kw OR ohca:ti,ab,kw OR ihca:ti,ab,kw OR 'post-arrest':ti,ab,kw OR postarrest:ti,ab,kw OR 'post cardiac arrest':ti,ab,kw OR 'post-cardiac arrest':ti,ab,kw OR postresuscitation:ti,ab,kw OR 'post-resuscitation':ti,ab,kw)

#2 Pediatric concept

**Search query**
('infant'/exp OR 'child'/exp OR 'adolescent'/exp OR 'pediatrics'/exp OR pediatric*:ti,ab,kw OR paediatric*:ti,ab,kw OR child*:ti,ab,kw OR infant*:ti,ab,kw OR adolescent*:ti,ab,kw)

#3 Temperature-management concept

**Search query**
('induced hypothermia'/exp OR 'body temperature regulation'/exp OR 'targeted temperature management':ti,ab,kw OR ttm:ti,ab,kw OR 'therapeutic hypothermia':ti,ab,kw OR 'induced hypothermia':ti,ab,kw OR 'mild hypothermia':ti,ab,kw OR hypothermia:ti,ab,kw OR cooling:ti,ab,kw OR normothermia:ti,ab,kw OR 'therapeutic normothermia':ti,ab,kw OR 'active normothermia':ti,ab,kw OR 'temperature management':ti,ab,kw OR 'temperature control':ti,ab,kw OR 'controlled temperature':ti,ab,kw OR 'temperature target':ti,ab,kw OR 'fever prevention':ti,ab,kw OR 'fever avoidance':ti,ab,kw OR fever:ti,ab,kw)

#4 Main combined search

**Search query**
('heart arrest'/exp OR 'cardiopulmonary resuscitation'/exp OR 'return of spontaneous circulation'/exp OR 'cardiac arrest':ti,ab,kw OR 'heart arrest':ti,ab,kw OR 'cardiopulmonary arrest':ti,ab,kw OR 'cardiorespiratory arrest':ti,ab,kw OR 'cardiopulmonary resuscitation':ti,ab,kw OR cpr:ti,ab,kw OR rosc:ti,ab,kw OR 'return of spontaneous circulation':ti,ab,kw OR 'return of circulation':ti,ab,kw OR ohca:ti,ab,kw OR ihca:ti,ab,kw OR 'post-arrest':ti,ab,kw OR postarrest:ti,ab,kw OR 'post cardiac arrest':ti,ab,kw OR 'post-cardiac arrest':ti,ab,kw OR postresuscitation:ti,ab,kw OR 'post-resuscitation':ti,ab,kw)<br>AND<br>('infant'/exp OR 'child'/exp OR 'adolescent'/exp OR 'pediatrics'/exp OR pediatric*:ti,ab,kw OR paediatric*:ti,ab,kw OR child*:ti,ab,kw OR infant*:ti,ab,kw OR adolescent*:ti,ab,kw)<br>AND<br>('induced hypothermia'/exp OR 'body temperature regulation'/exp OR 'targeted temperature management':ti,ab,kw OR ttm:ti,ab,kw OR 'therapeutic hypothermia':ti,ab,kw OR 'induced hypothermia':ti,ab,kw OR 'mild hypothermia':ti,ab,kw OR hypothermia:ti,ab,kw OR cooling:ti,ab,kw OR normothermia:ti,ab,kw OR 'therapeutic normothermia':ti,ab,kw OR 'active normothermia':ti,ab,kw OR 'temperature management':ti,ab,kw OR 'temperature control':ti,ab,kw OR 'controlled temperature':ti,ab,kw OR 'temperature target':ti,ab,kw OR 'fever prevention':ti,ab,kw OR 'fever avoidance':ti,ab,kw OR fever:ti,ab,kw)

No human limit was applied in the final search; animal-only records were excluded during screening.

#5 Supplemental duration validation search : 871 / 2026-08-17

Search query
('heart arrest'/exp OR 'cardiopulmonary resuscitation'/exp OR 'return of spontaneous circulation'/exp OR 'cardiac arrest':ti,ab,kw OR 'heart arrest':ti,ab,kw OR 'cardiopulmonary arrest':ti,ab,kw OR 'cardiorespiratory arrest':ti,ab,kw OR 'cardiopulmonary resuscitation':ti,ab,kw OR cpr:ti,ab,kw OR rosc:ti,ab,kw OR 'return of spontaneous circulation':ti,ab,kw OR 'return of circulation':ti,ab,kw OR ohca:ti,ab,kw OR ihca:ti,ab,kw OR 'post-arrest':ti,ab,kw OR postarrest:ti,ab,kw OR 'post cardiac arrest':ti,ab,kw OR 'post-cardiac arrest':ti,ab,kw OR postresuscitation:ti,ab,kw OR 'post-resuscitation':ti,ab,kw)<br>AND<br>('infant'/exp OR 'child'/exp OR 'adolescent'/exp OR 'pediatrics'/exp OR pediatric*:ti,ab,kw OR paediatric*:ti,ab,kw OR child*:ti,ab,kw OR infant*:ti,ab,kw OR adolescent*:ti,ab,kw)<br>AND<br>('induced hypothermia'/exp OR 'body temperature regulation'/exp OR 'targeted temperature management':ti,ab,kw OR ttm:ti,ab,kw OR 'therapeutic hypothermia':ti,ab,kw OR 'induced hypothermia':ti,ab,kw OR 'mild hypothermia':ti,ab,kw OR hypothermia:ti,ab,kw OR cooling:ti,ab,kw OR normothermia:ti,ab,kw OR 'therapeutic normothermia':ti,ab,kw OR 'active normothermia':ti,ab,kw OR 'temperature management':ti,ab,kw OR 'temperature control':ti,ab,kw OR 'controlled temperature':ti,ab,kw OR 'temperature target':ti,ab,kw OR 'fever prevention':ti,ab,kw OR 'fever avoidance':ti,ab,kw OR fever:ti,ab,kw)<br>AND<br>(duration:ti,ab,kw OR 'cooling duration':ti,ab,kw OR 'treatment duration':ti,ab,kw OR maintenance:ti,ab,kw OR '24 hour':ti,ab,kw OR '24 hours':ti,ab,kw OR '24 h':ti,ab,kw OR 24h:ti,ab,kw OR '48 hour':ti,ab,kw OR '48 hours':ti,ab,kw OR '48 h':ti,ab,kw OR 48h:ti,ab,kw OR '72 hour':ti,ab,kw OR '72 hours':ti,ab,kw OR '72 h':ti,ab,kw OR 72h:ti,ab,kw)

#6 Supplemental rewarming/maintenance search : 372 / 2026-08-17

Search query
('heart arrest'/exp OR 'cardiopulmonary resuscitation'/exp OR 'return of spontaneous circulation'/exp OR 'cardiac arrest':ti,ab,kw OR 'heart arrest':ti,ab,kw OR 'cardiopulmonary arrest':ti,ab,kw OR 'cardiorespiratory arrest':ti,ab,kw OR 'cardiopulmonary resuscitation':ti,ab,kw OR cpr:ti,ab,kw OR rosc:ti,ab,kw OR 'return of spontaneous circulation':ti,ab,kw OR 'return of circulation':ti,ab,kw OR ohca:ti,ab,kw OR ihca:ti,ab,kw OR 'post-arrest':ti,ab,kw OR postarrest:ti,ab,kw OR 'post cardiac arrest':ti,ab,kw OR 'post-cardiac arrest':ti,ab,kw OR postresuscitation:ti,ab,kw OR 'post-resuscitation':ti,ab,kw)<br>AND<br>('infant'/exp OR 'child'/exp OR 'adolescent'/exp OR 'pediatrics'/exp OR pediatric*:ti,ab,kw OR paediatric*:ti,ab,kw OR child*:ti,ab,kw OR infant*:ti,ab,kw OR adolescent*:ti,ab,kw)<br>AND<br>('induced hypothermia'/exp OR 'body temperature regulation'/exp OR 'targeted temperature management':ti,ab,kw OR ttm:ti,ab,kw OR 'therapeutic hypothermia':ti,ab,kw OR 'induced hypothermia':ti,ab,kw OR 'mild hypothermia':ti,ab,kw OR hypothermia:ti,ab,kw OR cooling:ti,ab,kw OR normothermia:ti,ab,kw OR 'therapeutic normothermia':ti,ab,kw OR 'active normothermia':ti,ab,kw OR 'temperature management':ti,ab,kw OR 'temperature control':ti,ab,kw OR 'controlled temperature':ti,ab,kw OR 'temperature target':ti,ab,kw OR 'fever prevention':ti,ab,kw OR 'fever avoidance':ti,ab,kw OR fever:ti,ab,kw)<br>AND<br>(rewarming:ti,ab,kw OR 're-warming':ti,ab,kw OR 'rewarming rate':ti,ab,kw OR maintenance:ti,ab,kw OR 'maintenance phase':ti,ab,kw)

S1D. Scopus

Platform: Scopus document search | Final strategy version: 2026-08-10 | Final execution date: 2026-08-17 | Results/exported: 3,250/3,250 records

Main combined search

Search query
TITLE-ABS-KEY(("cardiac arrest" OR "heart arrest" OR "cardiopulmonary arrest" OR "cardiorespiratory arrest" OR "cardiopulmonary resuscitation" OR CPR OR ROSC OR "return of spontaneous circulation" OR "return of circulation" OR OHCA OR IHCA OR "post-arrest" OR postarrest OR "post cardiac arrest" OR "post-cardiac arrest" OR postresuscitation OR "post-resuscitation")<br>AND<br>(pediatric* OR paediatric* OR child* OR infant* OR adolescent*)<br>AND<br>("targeted temperature management" OR TTM OR "therapeutic hypothermia" OR "induced hypothermia" OR "mild hypothermia" OR hypothermia OR cooling OR normothermia OR "therapeutic normothermia" OR "active normothermia" OR "temperature management" OR "temperature control" OR "controlled temperature" OR "temperature target" OR "fever prevention" OR "fever avoidance" OR fever))

Supplemental duration validation search : 647 / 2026-08-17

Search query
TITLE-ABS-KEY(("cardiac arrest" OR "heart arrest" OR "cardiopulmonary arrest" OR "cardiorespiratory arrest" OR "cardiopulmonary resuscitation" OR CPR OR ROSC OR "return of spontaneous circulation" OR "return of circulation" OR OHCA OR IHCA OR "post-arrest" OR postarrest OR "post cardiac arrest" OR "post-cardiac arrest" OR postresuscitation OR "post-resuscitation")<br>AND<br>(pediatric* OR paediatric* OR child* OR infant* OR adolescent*)<br>AND<br>("targeted temperature management" OR TTM OR "therapeutic hypothermia" OR "induced hypothermia" OR "mild hypothermia" OR hypothermia OR cooling OR normothermia OR "therapeutic normothermia" OR "active normothermia" OR "temperature management" OR "temperature control" OR "controlled temperature" OR "temperature target" OR "fever prevention" OR "fever avoidance" OR fever)<br>AND<br>(duration OR "cooling duration" OR "treatment duration" OR maintenance OR "24 hour" OR "24 hours" OR "24 h" OR 24h OR "48 hour" OR "48 hours" OR "48 h" OR 48h OR "72 hour" OR "72 hours" OR "72 h" OR 72h))

Supplemental rewarming/maintenance search : 297 / 2026-08-17

Search query
TITLE-ABS-KEY(("cardiac arrest" OR "heart arrest" OR "cardiopulmonary arrest" OR "cardiorespiratory arrest" OR "cardiopulmonary resuscitation" OR CPR OR ROSC OR "return of spontaneous circulation" OR "return of circulation" OR OHCA OR IHCA OR "post-arrest" OR postarrest OR "post cardiac arrest" OR "post-cardiac arrest" OR postresuscitation OR "post-resuscitation")<br>AND<br>(pediatric* OR paediatric* OR child* OR infant* OR adolescent*)<br>AND<br>("targeted temperature management" OR TTM OR "therapeutic hypothermia" OR "induced hypothermia" OR "mild hypothermia" OR hypothermia OR cooling OR normothermia OR "therapeutic normothermia" OR "active normothermia" OR "temperature management" OR "temperature control" OR "controlled temperature" OR "temperature target" OR "fever prevention" OR "fever avoidance" OR fever)<br>AND<br>(rewarming OR "re-warming" OR "rewarming rate" OR maintenance OR "maintenance phase"))

S1E. Web of Science Core Collection

Platform: Web of Science Advanced Search | Final strategy version: 2026-08-10 | Final execution date: 2026-08-20 | Results/exported: 1,039/1,039 records

Main combined search | Final execution: 2026-08-20 | Results/exported: 1,039/1,039 records

Search query
TS=(("cardiac arrest" OR "heart arrest" OR "cardiopulmonary arrest" OR "cardiorespiratory arrest" OR "cardiopulmonary resuscitation" OR CPR OR ROSC OR "return of spontaneous circulation" OR "return of circulation" OR OHCA OR IHCA OR "post-arrest" OR postarrest OR "post cardiac arrest" OR "post-cardiac arrest" OR postresuscitation OR "post-resuscitation")<br>AND<br>(pediatric* OR paediatric* OR child* OR infant* OR adolescent*)<br>AND<br>("targeted temperature management" OR TTM OR "therapeutic hypothermia" OR "induced hypothermia" OR "mild hypothermia" OR hypothermia OR cooling OR normothermia OR "therapeutic normothermia" OR "active normothermia" OR "temperature management" OR "temperature control" OR "controlled temperature" OR "temperature target" OR "fever prevention" OR "fever avoidance" OR fever))

Supplemental duration validation search 261

Search query
TS=(("cardiac arrest" OR "heart arrest" OR "cardiopulmonary arrest" OR "cardiorespiratory arrest" OR "cardiopulmonary resuscitation" OR CPR OR ROSC OR "return of spontaneous circulation" OR "return of circulation" OR OHCA OR IHCA OR "post-arrest" OR postarrest OR "post cardiac arrest" OR "post-cardiac arrest" OR postresuscitation OR "post-resuscitation")<br>AND<br>(pediatric* OR paediatric* OR child* OR infant* OR adolescent*)<br>AND<br>("targeted temperature management" OR TTM OR "therapeutic hypothermia" OR "induced hypothermia" OR "mild hypothermia" OR hypothermia OR cooling OR normothermia OR "therapeutic normothermia" OR "active normothermia" OR "temperature management" OR "temperature control" OR "controlled temperature" OR "temperature target" OR "fever prevention" OR "fever avoidance" OR fever)<br>AND<br>(duration OR "cooling duration" OR "treatment duration" OR maintenance OR "24 hour" OR "24 hours" OR "24 h" OR 24h OR "48 hour" OR "48 hours" OR "48 h" OR 48h OR "72 hour" OR "72 hours" OR "72 h" OR 72h))

Supplemental rewarming/maintenance search 122

Search query
TS=(("cardiac arrest" OR "heart arrest" OR "cardiopulmonary arrest" OR "cardiorespiratory arrest" OR "cardiopulmonary resuscitation" OR CPR OR ROSC OR "return of spontaneous circulation" OR "return of circulation" OR OHCA OR IHCA OR "post-arrest" OR postarrest OR "post cardiac arrest" OR "post-cardiac arrest" OR postresuscitation OR "post-resuscitation")<br>AND<br>(pediatric* OR paediatric* OR child* OR infant* OR adolescent*)<br>AND<br>("targeted temperature management" OR TTM OR "therapeutic hypothermia" OR "induced hypothermia" OR "mild hypothermia" OR hypothermia OR cooling OR normothermia OR "therapeutic normothermia" OR "active normothermia" OR "temperature management" OR "temperature control" OR "controlled temperature" OR "temperature target" OR "fever prevention" OR "fever avoidance" OR fever)<br>AND<br>(rewarming OR "re-warming" OR "rewarming rate" OR maintenance OR "maintenance phase"))

S1F. Cochrane CENTRAL

Platform: Cochrane Library Search Manager | Final strategy version: 2026-08-10 | Final execution date: 2026-08-17 | Results/exported: 168/168 CENTRAL records

Line	Exact query
#1	MeSH descriptor: [Heart Arrest] explode all trees
#2	MeSH descriptor: [Out-of-Hospital Cardiac Arrest] explode all trees
#3	MeSH descriptor: [Cardiopulmonary Resuscitation] explode all trees
#4	("cardiac arrest" OR "heart arrest" OR "cardiopulmonary arrest" OR "cardiorespiratory arrest" OR "cardiopulmonary resuscitation" OR CPR OR ROSC OR "return of spontaneous circulation" OR "return of circulation" OR OHCA OR IHCA OR "post-arrest" OR postarrest OR "post cardiac arrest" OR "post-cardiac arrest" OR postresuscitation OR "post-resuscitation"):ti,ab,kw
#5	#1 OR #2 OR #3 OR #4
#6	MeSH descriptor: [Infant] explode all trees
#7	MeSH descriptor: [Child] explode all trees
#8	MeSH descriptor: [Adolescent] explode all trees
#9	MeSH descriptor: [Pediatrics] explode all trees
#10	(pediatric OR paediatric OR child OR children OR infant OR infants OR adolescent OR adolescents):ti,ab,kw
#11	#6 OR #7 OR #8 OR #9 OR #10
#12	MeSH descriptor: [Hypothermia, Induced] explode all trees
#13	MeSH descriptor: [Body Temperature Regulation] explode all trees
#14	("targeted temperature management" OR TTM OR "therapeutic hypothermia" OR "induced hypothermia" OR "mild hypothermia" OR hypothermia OR cooling OR normothermia OR "therapeutic normothermia" OR "active normothermia" OR "temperature management" OR "temperature control" OR "controlled temperature" OR "temperature target" OR "fever prevention" OR "fever avoidance" OR fever):ti,ab,kw
#15	#12 OR #13 OR #14
#16 // n = 168	#5 AND #11 AND #15

Line	Exact query
#17 // n = 93	#16 AND (duration OR "cooling duration" OR "treatment duration" OR maintenance OR "24 hour" OR "24 hours" OR "24 h" OR 24h OR "48 hour" OR "48 hours" OR "48 h" OR 48h OR "72 hour" OR "72 hours" OR "72 h" OR 72h):ti,ab,kw
#18 // n = 23	#16 AND (rewarming OR "re-warming" OR "rewarming rate" OR maintenance OR "maintenance phase"):ti,ab,kw

Use #16 as the main CENTRAL screening set. Lines #17 and #18 are supplemental validation searches only.

S1G. ClinicalTrials.gov

Platform: ClinicalTrials.gov Expert Search | Final strategy version: 2026-08-10 | Final execution date: 2026-08-17 | Results/exported: 72/72 study records

Broad combined registry search

**Search query**
("cardiac arrest" OR "heart arrest" OR "cardiopulmonary arrest" OR "cardiorespiratory arrest" OR "cardiopulmonary resuscitation" OR CPR OR ROSC OR "return of spontaneous circulation" OR "return of circulation" OR OHCA OR IHCA OR "post-arrest" OR postarrest OR "post cardiac arrest" OR "post-cardiac arrest" OR postresuscitation OR "post-resuscitation")<br>AND<br>(pediatric OR paediatric OR child OR children OR infant OR infants OR adolescent OR adolescents)<br>AND<br>("targeted temperature management" OR TTM OR "therapeutic hypothermia" OR "induced hypothermia" OR "mild hypothermia" OR hypothermia OR cooling OR normothermia OR "therapeutic normothermia" OR "active normothermia" OR "temperature management" OR "temperature control" OR "controlled temperature" OR "temperature target" OR "fever prevention" OR "fever avoidance" OR fever)

Search all study statuses and study types. Do not impose an additional age or date filter unless required by the interface; assess eligibility during screening.
